# Oral Microbiome and Gingival Tissue Apoptosis and Autophagy Transcriptomics

**DOI:** 10.3389/fimmu.2020.585414

**Published:** 2020-10-19

**Authors:** Jeffrey L. Ebersole, Sreenatha S. Kirakodu, Elliot Neumann, Luis Orraca, Janis Gonzalez Martinez, Octavio A. Gonzalez

**Affiliations:** ^1^Department of Biomedical Science, School of Dental Medicine, University of Nevada Las Vegas, Las Vegas, NV, United States; ^2^Center for Oral Health Research, University of Kentucky, Lexington, KY, United States; ^3^Division of Periodontology, College of Dentistry, University of Kentucky, Lexington, KY, United States; ^4^School of Dental Medicine, University of Puerto Rico, San Juan, Puerto Rico; ^5^Caribbean Primate Research Center, University of Puerto Rico, San Juan, Puerto Rico

**Keywords:** non-human primates, apoptosis, autophagy, microbiome, periodontitis

## Abstract

**Objective:** This study focused on documenting characteristics of the gingival transcriptome during various stages of periodontitis targeting genes associated with apoptotic and autophagic pathways and changes that specifically associate with features of the oral microbiome.

**Methods:** *Macaca mulatta* (*n* = 18; 12–23 years) were examined at baseline and 0.5, 1, and 3 months of disease progression, as well as 5 months with clinical disease resolution. 16S sequencing and microarray analyses examined changes in the microbiome and gingival transcriptome, respectively, at each time point from every animal.

**Results:** Specific patterns of apoptotic and autophagic genes were identified related to the initiation and progression of disease. The analysis also provided insights on the principal bacteria within the complex microbiome whose abundance was significantly correlated with differences in apoptotic and autophagic gene expression. Bacteria were identified that formed associated complexes with similar effects on the host gene expression profiles. A complex of *Leptotrichia*_unclassifed, *Capnocytophaga*_unclassified, *Prevotella* sp. 317, and *Veillonellaceae*_[G-1] sp. 155 were significantly negatively correlated with both apoptosis and autophagy. Whereas, *Veillonellaceae*_[G-1], *Porphyromonadaceae*, and *F. alocis* 539 were significantly positively correlated with both pathways, albeit this relationship was primarily associated with pro-apoptotic genes.

**Conclusions:** The findings provide evidence for specific bacteria/bacterial complexes within the oral microbiome that appear to have a more substantive effect on regulating apoptotic and autophagic pathways in the gingival tissues with periodontitis.

Apoptosis and autophagy are described as important regulators of cellular functions, particularly related to nutritional stress that could be induced by infection and inflammation. As such, recent results support an important role for each of these cell death or survival pathways in regulating inflammatory and immune responses. Periodontitis represents a complex chronic microbial challenge by a dysbiotic microbiome that results in a persistent inflammatory response and a tissue destructive immunoinflammatory lesion. Although there are clear clinical symptoms of this disease including soft and hard tissue destruction of the periodontium, the underlying biology of the host-bacterial interactions that lead to initiation, progression, and resolution of the disease remain less clear. We have used a non-human primate model of ligature-induced experimental periodontal disease to try to model these host-bacterial interactions.

## Introduction

Mucosal surfaces of the body, including the oral cavity, are continually interacting with a complex microbiome (1). This challenge results in activation of an array of immune response pathways, and cells and biomolecules that maintain homeostasis. Moreover, the oral cavity presents a model of mucosal host-bacterial interactions whereby specific microorganisms colonize various niches (2) creating complex biofilms that change with local environmental cues and respond to disease processes of the periodontium (3–6).

Chronic periodontitis reflects an active breakdown of connective tissue and resorption of alveolar bone, with localized lesions attributed to a dysregulated host inflammatory and immune responses to dysbiotic microbial biofilms (7–12). Microbiome changes with disease have been shown to include alterations in specific members of the subgingival microbiome at disease sites (e.g., *Porphyromonas gingivalis*) with the capacity to alter the biologic activities of the overall microbiome, as well as regulating host responses that would contribute to the tissue breakdown as a hallmark of periodontitis (13, 14).

The gingival mucosal tissues are enriched in cellular turnover with many cells undergoing programmed cell death (*i.e.*, apoptosis) (15). Apoptotic processes appear important for natural maintenance of an intact epithelial barrier that contributes anti-microbial resistance (16) and appears to be an important mechanism that regulates the immunoinflammatory response to microbial challenge (15, 17, 18). Moreover, autophagy is a well-described mechanism for host cells to engulf microbes or damaged cell material into autophagosomes for eventual degradation (19). Cells with defective autophagy pathways exhibit exaggerated inflammation and increased susceptibility to infections (20–23). Additionally, various microbial species appear to modulate autophagy as a virulence strategy to enable persistent survival inside host cells affecting both anti-microbial and anti-inflammatory responses (24, 25).

The characterization of both apoptosis and autophagy as having important roles in infection and inflammation is a relatively recent concept. The impact of these cellular functions on anti-microbial and anti-inflammatory properties suggests that alterations could contribute to the pathogenesis of periodontitis. Existing data indicates that specific bacteria within the oral ecology produce components that inhibit apoptotic pathways (26–28) and can modulate autophagic responses, including *P. gingivalis* (26, 29, 30). These functions have been described to enable evasion of responses and persistent infection of the oral epithelium, and even enhance survival inside endothelial cells (31). Knowledge of the role of apoptosis and autophagy in periodontitis remains rather limited and thus studies of these pathways in mucosal tissues related to gingival health and periodontitis are clearly needed.

We have described gene expression profiles in gingival tissues from young to aged non-human primates, *Macaca mulatta*, and demonstrated altered patterns of apoptotic (32, 33) and autophagic (34) pathway genes affected by aging and periodontitis. Imbalances in these processes would be consistent with the capacity to respond to the microbial challenge, as well as a disruption related to the disease process (35–37). This study focused on the analysis of the expression of targeted gene sets related to the pathways of apoptosis and various phases of autophagy pathways using the non-human primate model of progressing periodontitis. The gingival transcriptome expression was specifically integrated into a model exploring the relationship between characteristics of the oral microbiome matched to gingival sites in health and progressing disease.

## Materials and Methods

### Animals and Diet

Rhesus monkeys (*Macaca mulatta*) (*n* = 18; 12–23 years of age) housed at the Caribbean Primate Research Center at Sabana Seca, Puerto Rico were examined for periodontal health or naturally-occurring periodontitis (32, 33, 38). The non-human primates were fed a 20% protein, 5% fat, and 10% fiber commercial monkey diet (diet 8773, Teklad NIB primate diet modified: Harlan Teklad, Madison, WI). The diet was supplemented with fruits and vegetables, and water was provided *ad libitum* in an enclosed corral setting.

**Figure 1:**
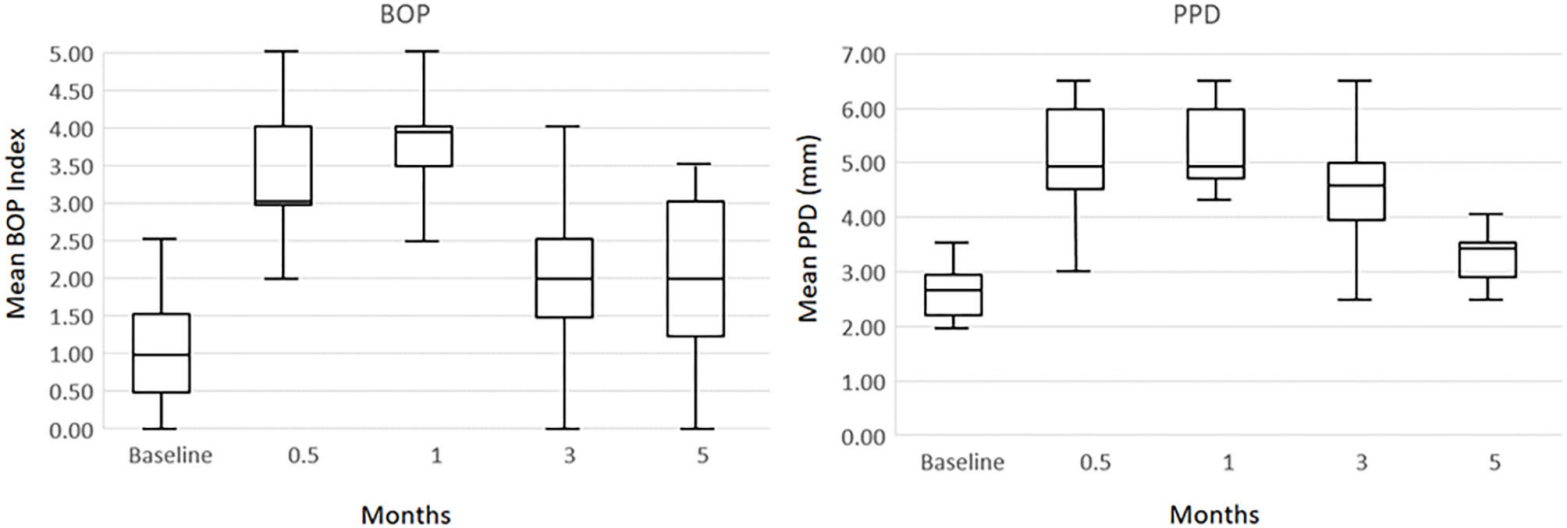
Boxplot of clinical measures of ligated teeth in the 18 animals at each time point for bleeding on probing (BOP) index and probing pocket depth (PPD).

As we have reported previously the protocol was approved by the Institutional Animal Care and Use Committee (IACUC) of the University of Puerto Rico and a ligature disease model was utilized (39). The clinical examination included probing pocket depth (PPD) and bleeding on probing (BOP; 0–5 scale) (40). Periodontal health was defined by mean Pocket Depth (PD) ≤ 3.0 mm and mean Bleeding on Probing (BOP) ≤ 1 (0–5 scale) in a full mouth examination excluding 3rd molars and canines (39). Ligature-induced periodontal disease was initiated as we have previously reported and clinical changes were compared to baseline measures of all maxillary and mandibular premolars and 1st and 2nd molars that were then ligated. The ligature-induced periodontitis model was implemented as we have described previously, by tying 3-0 silk sutures around the necks of maxillary and mandibular premolar and 1st and 2nd molar teeth in each animal (41, 42). As noted previously, removal of ligatures leads to a decrease in inflammation and BOP to normal levels and stabilization of any pocket probing depths in the non-human primates (42). Gingiva tissue biopsies and subgingival plaque samples taken at 0.5, 1, and 3 months (Initiation/Progression), and 2 months after removal of ligatures and local factors (Resolution). Determination of periodontal disease at the sampled site was documented by assessment of the presence of BOP and probing pocket depth of > 4 mm, as we have described previously (33). Figure 1 presents the clinical findings for both bleeding on probing and probing pocket depth for the 18 animals. As shown previously, these clinical measures of periodontal inflammation and tissue changes occur as early as 0.5 months post-ligature (Baseline), and progress through the 3 months of disease progression.

### Microbiome Analysis

Subgingival bacterial samples were obtained from the 18 animals by a curette and analyzed using a MiSeq instrument (43, 44) for the total composition of the microbiome from each sample (45, 46). Sequences were clustered into phylotypes based on their sequence similarity and these binned phylotypes were assigned to their respective taxonomic classification using the Human Oral Microbiome Database (HOMD V13) (http://www.homd.org/index.php?name=seqDownload&file&type=R) as we have described previously (43). Raw data were deposited at the NIH NCBI (BioProject ID PRJNA516659). Statistical differences of bacterial OTUs were determined with a *t-test* (*p* < 0.05). Correlations of OTUs within the oral microbiome were determined using a Pearson correlation coefficient analysis (*p* < 0.05). Correlations between the microbiome components and the gingival gene expression were determined only for matching samples derived from the same tooth in each of the animals. Matching samples with sufficient microbiome signals were compared for 58 samples obtained throughout the ligature model.

### Gingival Tissue Sample Collection and mRNA Analysis

Gingival tissue samples of healthy and disease sites were surgically collected and total RNA extracted for microarray analysis via hybridization to the GeneChip® Rhesus Gene 1.0 ST Array (Affymetrix, Santa Clara, CA, USA) similar to methods we have described previously (32, 33, 47–49).

### Data Analysis

Pro- and anti-apoptosis pathway genes (*n* = 27) and 33 genes involved in the autophagy pathway (Table 1) that we had previously identified in non-human primates as being affected by age or periodontitis were targeted. The expression intensities across the samples were estimated using the Robust Multi-array Average (RMA) algorithm with probe-level quintile normalization, as implemented in the Partek Genomics Suite software version 6.6 (Partek, St. Louis, MO). The different groups were initially compared using one way ANOVA. For genes that had significant mean differences, two sample *t*-tests were used to investigate differences. Statistical significance was considered by a *p* ≤ 0.05. Correlation analyses between the microbiome Otus and the gingival gene expression were performed using a Spearman rank-order correlation analysis. The data has been uploaded into the ArrayExpress data base (www.ebi.ac.uk) under accession number: E-MTAB-1977.

**Table 1:** Listing of host genes examined for apoptosis and autophagy processes in the gingival tissues.

**Gene ID**	**Gene name**	**Fxn**	**Gene ID**	**Gene name**	**Fxn**
**Apoptosis**			**Autophagy**		
AKT3	AKT Serine/Threonine Kinase 3	A	EIF4G1	Eukaryotic Translation Initiation Factor 4 Gamma 1	MTOR
BIRC3	Baculoviral IAP Repeat Containing 3	A	MAPKSP1/LAMTOR3	Late Endosomal/Lysosomal Adaptor, MAPK and, MTOR Activator 3	MTOR
CD2	CD2 Molecule	A	ATG101	Autophagy Related 101	ULK
CFLAR	CASP8 and FADD Like Apoptosis Regulator	A	DRAM1	DNA Damage Regulated Autophagy Modulator 1	ULK
CSF2RB	Colony Stimulating Factor 2 Receptor Subunit Beta	A	DRAM2	DNA Damage Regulated Autophagy Modulator 2	ULK
NOL3	Nucleolar Protein 3	A	SNX4	Sorting Nexin 4	ULK
PIK3CD	Phosphatidylinositol-4,5-Bisphosphate 3-Kinase Catalytic Subunit Delta	A	ATG14/Barkor	Autophagy Related 14	PI3K
PIK3CG	Phosphatidylinositol-4,5-Bisphosphate 3-Kinase Catalytic Subunit Gamma	A	DAPK1	Death Associated Protein Kinase 1	PI3K
PRKACB	Protein Kinase CAMP-Activated Catalytic Subunit Beta	A	IGF1	Insulin Like Growth Factor 1	PI3K
APAF1	Apoptotic Peptidase Activating Factor 1	P	PIK3CG	Phosphatidylinositol-4,5-Bisphosphate 3-Kinase Catalytic Subunit Gamma	PI3K
ATM	ATM Serine/Threonine Kinase	P	RB1	RB Transcriptional Corepressor 1	PI3K
BID	BH3 Interacting Domain Death Agonist	P	SHIP2/INPPL1	Inositol Polyphosphate Phosphatase Like 1	PI3K
CASP1	Caspase 1	P	ATG3	Autophagy Related 3	ATG12
CASP3	Caspase 3	P	ATG4C	Autophagy Related 4C	ATG12
CASP7	Caspase 7	P	ATG4D	Autophagy Related 4D	ATG12
CASP8	Caspase 8	P	ATG5	Autophagy Related 5	ATG12
CASP10	Caspase 10	P	ATG7	Autophagy Related 7	ATG12
DAPK1	Death Associated Protein Kinase 1	P	ATG16L2	Autophagy Related Like 2	ATG12
ENDOD1	Endonuclease Domain Containing 1	P	CALCOCO2	Calcium Binding and Coiled-Coil Domain 2	ATG12
FAS	Fas Cell Surface Death Receptor	P	CXCR4	C-X-C Motif Chemokine Receptor 4	ATG12
IL1A	Interleukin-1 Alpha	P	EIF2AK3/PERK	Eukaryotic Translation Initiation Factor 2 Alpha Kinase 3	ATG12
IL1B	Interleukin-1 Beta	P	EIF2AK4/GCN2	Eukaryotic Translation Initiation Factor 2 Alpha Kinase 4	ATG12
IL1R1	Interleukin 1 Receptor Type 1	P	GABARAPL2/ ATG8c	GABA Type A Receptor Associated Protein Like 2	ATG12
IL1RAP	Interleukin 1 Receptor Accessory Protein	P	PLIN2	Perilipin 2	ATG12
IRAK3	Interleukin 1 Receptor Associated Kinase 3	P	BAD	BCL2 Associated Agonist Of Cell Death	LF/VD
PRKAR2B	Protein Kinase CAMP-Dependent Type II Regulatory Subunit Beta	P	BAK1	BCL2 Antagonist/Killer 1	LF/VD
TNFRS11B	TNF Receptor Superfamily Member 11b	P	CASP8	Caspase 8	LF/VD
TRAF3	TNF Receptor Associated Factor 3	P	CTSL2/CTSV	Cathepsin V	LF/VD
			EPAS1	Endothelial PAS Domain Protein 1	LF/VD
			FAS	Fas Cell Surface Death Receptor	LF/VD
			LAMP2	Lysosomal Associated Membrane Protein 2	LF/VD
			PRKCQ	Protein Kinase C Theta	LF/VD
			VAMP8	Vesicle Associated Membrane Protein 8	LF/VD

*Functions denote: anti-apoptosis (A), pro-apoptosis (P), mTORC1 complex (mTOR), ULK complex (ULK), PI3K complex (PI3K), ATG12 interactions (ATG12), and lysosome fusion/vesicle degradation (LF/VD)*.

## Results

### Longitudinal Changes in Apoptosis Gene Expression Profiles

**Figure 2:**
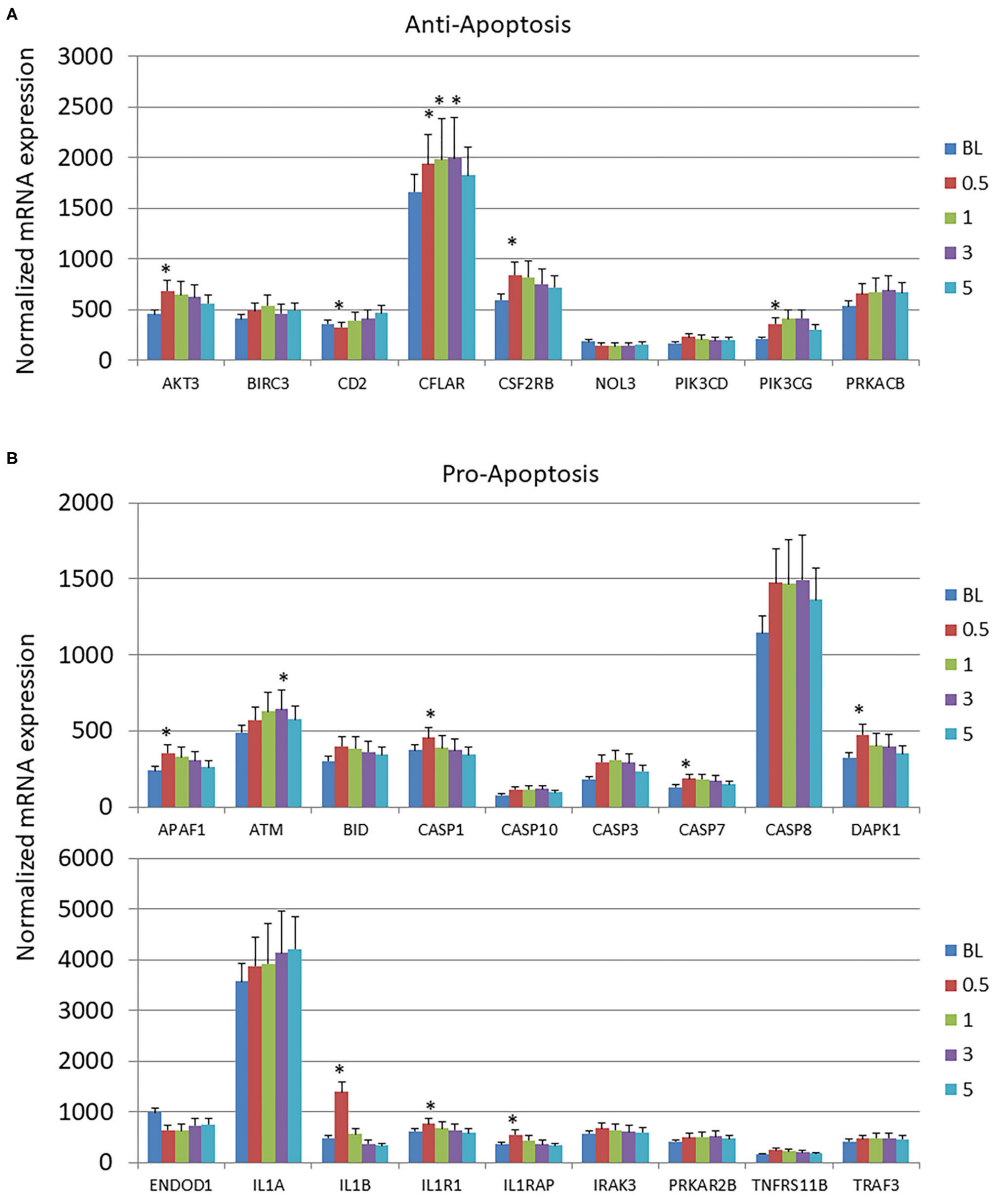
Expression levels of **(A)** anti-apoptotic and **(B)** pro-apoptotic genes in gingival samples at baseline (BL) and 0.5, 1, and 3 months following ligation. Five month samples represent 60 days following removal of the ligatures with resolution of the clinical features of the disease. The bars denote the means of 18 animal samples and the vertical bracket signifies one SD. The asterisk (*) denotes significantly different from baseline levels at *p* < 0.05.

Few changes from baseline healthy tissues were observed in the anti-apoptosis genes across the disease process or with resolution (Figure 2A). In contrast, Figure 2B summarizes that 8/18 of the pro-apoptosis genes were significantly altered through the disease process. The majority of these changes were increases occurring at initiation of disease.

### Longitudinal Changes in Autophagy Gene Expression Profiles

**Figure 3:**
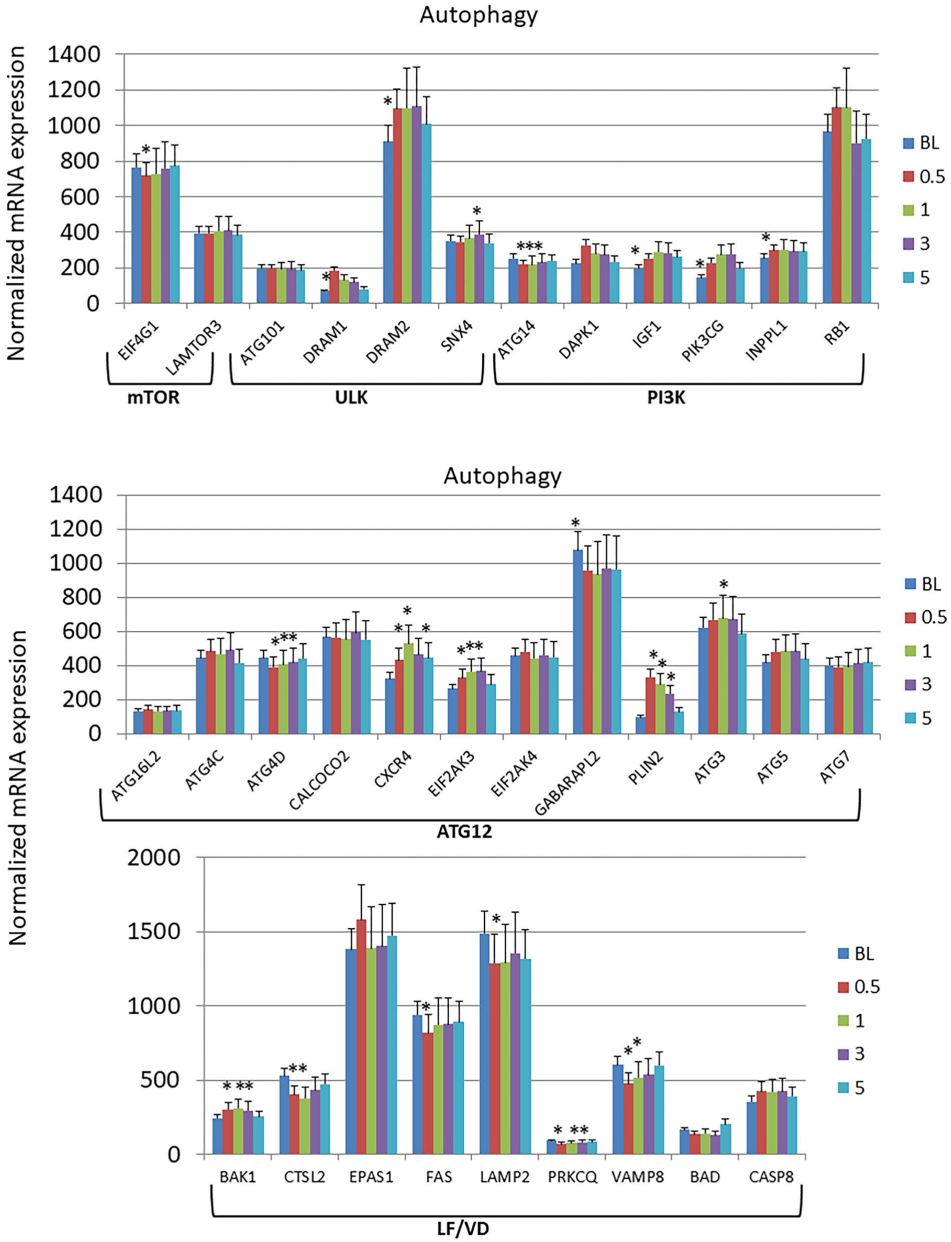
Expression levels of autophagy genes in gingival samples at baseline (BL) and 0.5, 1, and 3 months following ligation. Five month samples represent 60 days following removal of the ligatures with resolution of the clinical features of the disease. The genes are grouped into various categories representing steps in the autophagy pathway. The bars denote the means of 18 animal samples and the vertical bracket signifies one SD. The asterisk (*) denotes significantly different from baseline levels or baseline levels being different from all other time points at *p* < 0.05.

The autophagy gene expression profiles were organized into 6 phases of the process including initial interactions through the Mechanistic Target Of Rapamycin Kinase (mTOR) and Unc-51 Like Autophagy Activating Kinase 1 (ULK) complexes, signaling activities via genes in the PI3K (Phosphatidylinositol-4,5-Bisphosphate 3-Kinase) complex, expansion of the phagophore via Autophagy Related 12 (ATG12)-related genes, and finally linked to lysosome formation and vesicle degradation (LF/VD) processes (Figure 3). Generally in the early stages of the process (mTOR, ULK, PI3K), gene expression alterations were increased from baseline health to initiation and progression of disease. A larger number of gene expression level changes were identified in the ATG12 portion of the autophagy pathway with most demonstrating elevated levels compared to health (baseline). Only ATG4D and GABA Type A Receptor Associated Protein Like 2 (GABARAPL2; ATG8C) levels were decreased with disease. Finally, LF/VD gene expression appeared to be decreased during disease.

### Microbiome Members Associated With Altered Apoptosis and Autophagy Gene Expression

**Figure 4:**
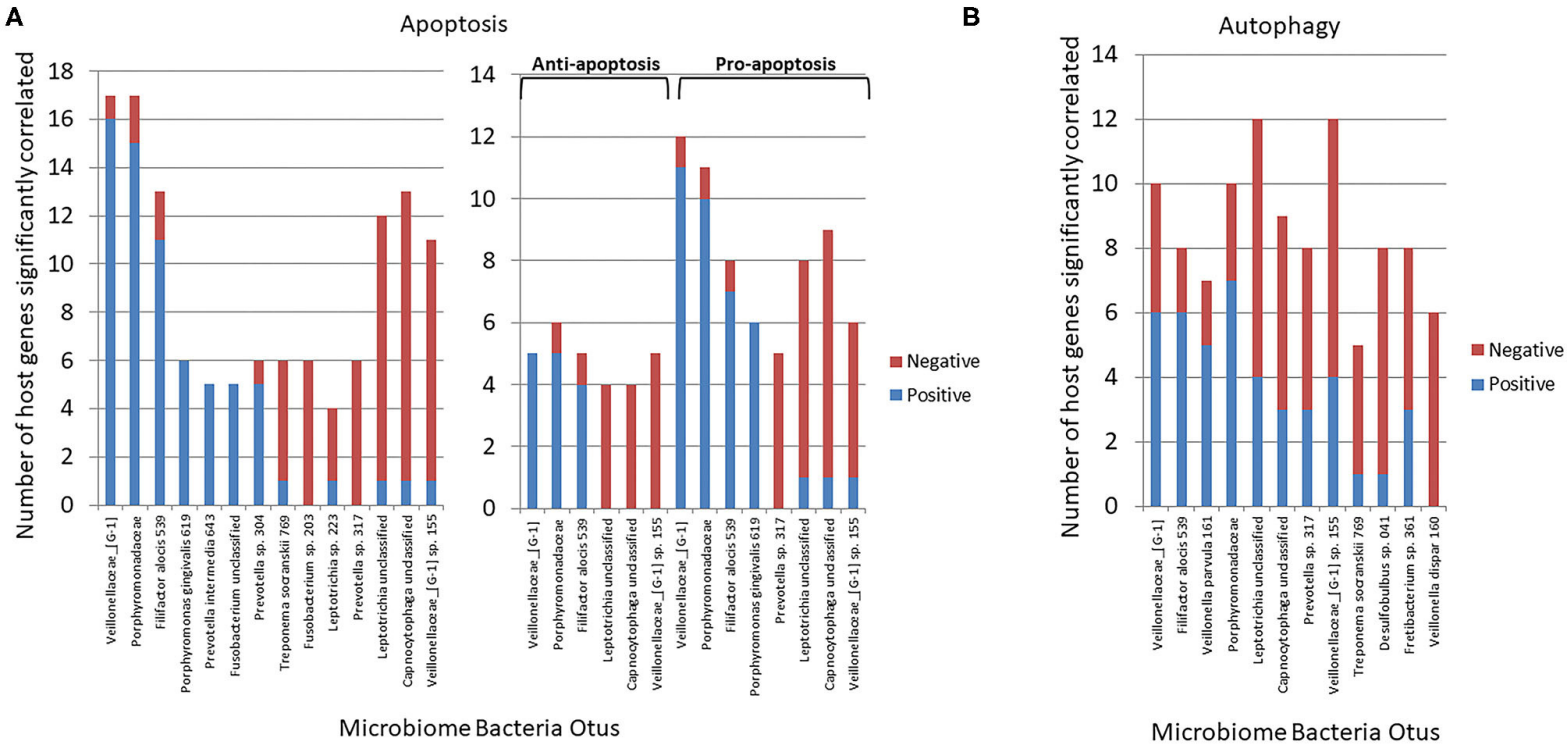
Correlations (*p* < 0.05) between individual bacterial abundance within the microbiome to gene expression levels from the same sites. **(A)** Frequency of correlations for all the apoptosis genes for each bacterial OTU and stratified based upon significant correlations specifically with anti-apoptosis or pro-apoptosis genes. **(B)** Frequency of correlations for all autophagy genes for each bacterial OTU.

Figure 4A summarizes the correlation patterns of the 22 apoptosis genes with specific bacteria among the 58 OTUs that account for 88% of the total microbial reads across the adult samples (43). Specific OTUs were significantly correlated with a high number of host genes, and displayed as ones with a majority of positive or negative correlations (Supplementary Table 1). The data also show that most of these OTUs had a predilection for either positive or negative correlations with the array of apoptotic genes. Also of note was that in all comparisons the correlations of these bacteria predominated with expression of pro-apoptotic genes either positively (*Veillonellaceae*_[G1], *Porphyromonadaceae, F. alocis* 539) or negatively (*Prevotella* sp. 317, *Leptotrichia* unclassified, *Capnocytophaga* unclassified). Figure 4B presents the relationship of the OTUs to the autophagy genes. While the bacteria (e.g., OTUs) generally showed both positive and negative correlations to genes within the complex of the autophagy pathway, as with apoptosis the individual OTUs were skewed toward a higher frequency of positive or negative correlations (Supplementary Table 2). Of interest was a considerable overlap in the specific OTUs of autophagy and apoptosis gene correlations, with ~2/3 of the OTUs overlapping.

**Figure 5:**
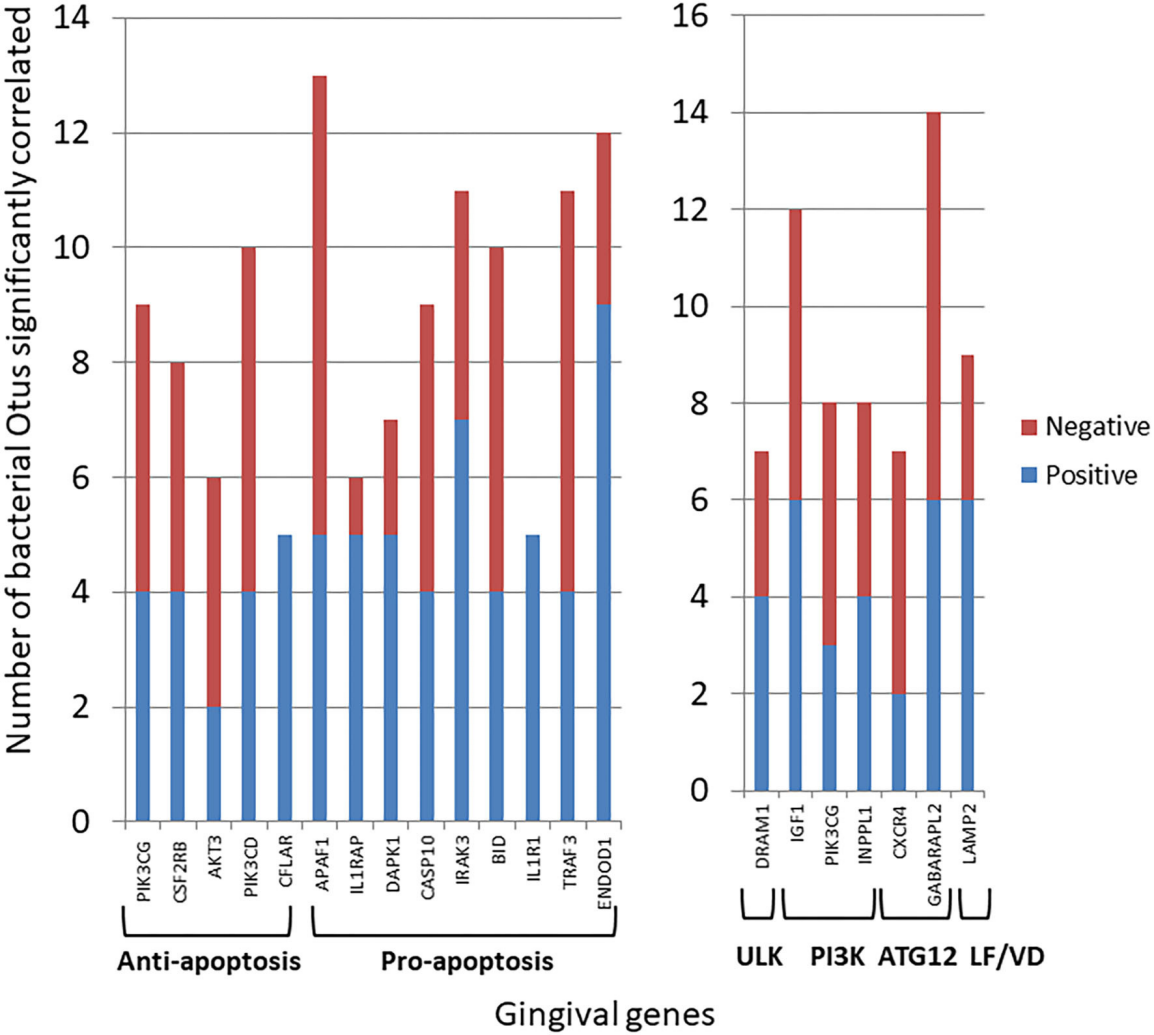
Individual gene expression levels correlated with bacterial abundance levels in the microbiome samples. The stacked bars depict the frequency of microbial correlations of a particular apoptotic or autophagy gene separated into significant (*p* < 0.05) positive or negative correlations. Each gene is organized into apoptosis processes or steps in the autophagy pathway. The genes presented had > 7 total bacterial correlations except for CFLAR and IL1R1 that showed a unique pattern of only positive correlations.

Transposing these data provided insight into the primary genes within the apoptosis or autophagy pathways demonstrating preferential correlations with the microbiome members across all the samples (Figure 5). These included both anti- and pro-apoptotic genes with most showing a similar number of bacteria exhibiting positive or negative correlations. A more limited number of autophagy genes across the pathway phases showed significant correlations with a larger number of individual bacteria, and as with apoptosis, there was a similar distribution of positively, and negatively correlated bacteria.

### Bacterial Complexes Related to Altered Apoptosis and Autophagy Gene Expression Profiles

Based upon the observation that there were multiple bacterial-host gene correlations for both apoptosis and autophagy, we explored the potential that complexes of certain bacteria demonstrated similar patterns of interaction with host responses across the disease model. Table 2 shows 3 bacterial complexes (AP-1 to AP-3) that were related in their frequency of correlations with a specific portfolio of anti- or pro-apoptotic genes. As noted, the same complex could show both significantly positive and negative correlations to these panels of different host genes. Table 3 provides a similar summary where 5 complexes (AU-1 to AU-5) that were identified to show significant correlations with multiple host genes across the autophagy pathway. Interestingly, there was not only considerable overlap in the complex members identified related to apoptosis and autophagy gene expression, but there were similarities in the specific complex groupings correlated with the gene expression levels. This was demonstrated by representatives in the AP-1/AU-2 complexes, AP-2/AU-1 complexes, and AP-3/AU-5 complexes.

**Table 2:** Complexes of bacteria with related significant correlations to apoptosis genes.

**ID**	**Bacterial complex**	**Anti**	**Pro**
AP-I	Veillonellaceae_[G-1] Porphyromonadaceae Filifactor alocis 539 Porphyromonas gingivalis 619 Prevotella intermedia 643 Fusobacterium unclassified *Prevotella* sp. 304	PI3KCG CSF2RB AKT3 PIK3CD CFLAR NOL3	IL1B TNFRS118 APAFl ILIRAP CASP7 DAPKI IRAK3 BID IL1R1 TRAF3 ENDOD1
AP-2	*Desulfobulbus* sp. 041 Treponema socranskii 769 *Fusobacterium* sp. 203 *Leptotrichia* sp. 223 Selenomonas unclassified *Prevotella* sp. 317 Leptotrichia uncla ssified Capnocytophaga unclassified *Veillonellaceae_[G-l]* sp. 155	PI3KCG CSF2RB AKT3 PIK3CD	CASP3 APAF1 CASP10 IRAK3 BID TRAF3 ENDOD1
AP-3	Porphyromonadaceae Prevotella intermedia 643 *Prevotella* sp. 313 Veillonella dispar 160		FAS

*The genes in Green are positively correlated and in Red are negatively correlated with the bacterial complexes*.

**Table 3:** Complexes of bacteria with related significant correlations to autophagy genes.

**ID**	**Bacterial complex**	**mTOR**	**ULK**	**PI3K**	**ATG9**	**ATG12**	**LF/VD**
AU-I	Leptotrichia unclassified Capnocytophaga unclassified *Prevotella* sp. 317 *Veillonellaceae_[G-1]* sp. 155 Treponema socranskii 769 *Desulfobulbus* sp. 041	EIF4G1	DRAM2	IGF1 PIK3CG DAPK1 PIK3CG INPPL1		ATG4D CXCR4 ElF2AK3 GABARAPl2 PllN2	LAMP2
AU-2	Veillonellaceae_[G-1] Fusobacterium unclassified Filifactor alocis 539		DRAM1				
AU-3	Prevotella fusca 782 *Prevotella* sp. 304 *SR1_[G-1]* sp. 345 Prevotella unclassified Pyramidobacter piscolens 357 Bacteria_unclassified		SNX4				
AU-4	Fretibacterium fastidiosum 363 Bacteroidetes_unclassified *Chloroflexi_[G-1]* sp. 439 *Fretibacterium* sp. 361		ATG101			GABARAPL2	
AU-5	Porphyromonadaceae Prevotella intermedia 643 *Prevotella* sp. 313 Veillonella dispar 160	EIF4G1	ATG101			ElF2AK4	EPAS1 FAS

*The genes in Green are positively correlated and in Red are negatively correlated with the bacterial complexes*.

### Bacterial Complexes Affecting Apoptosis and Autophagy Pathways

**Figure 6:**
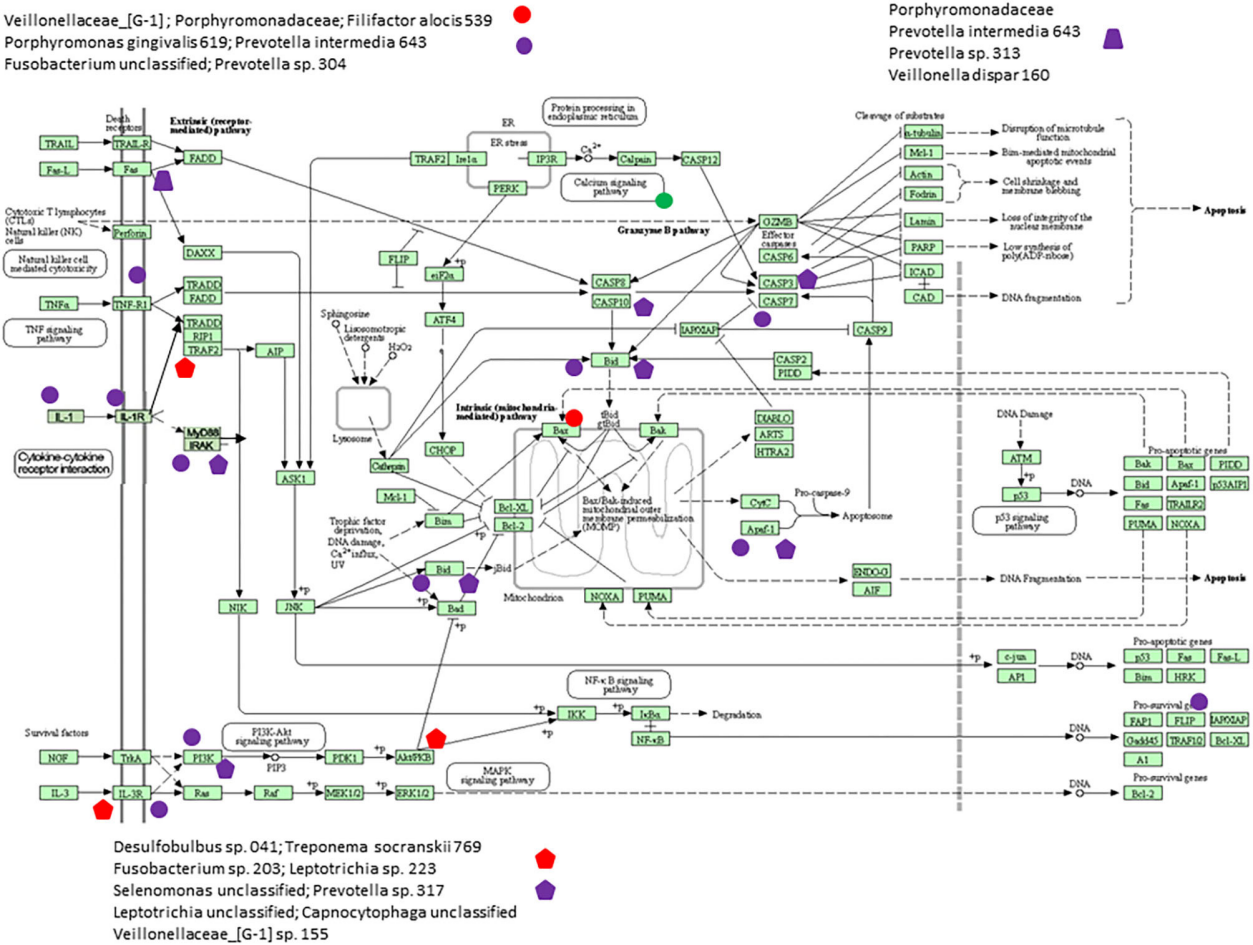
KEGG pathway graphic for apoptosis. The bacterial complexes are identified with effects on apoptosis genes with the red colored symbols denoting a negative correlation and the purple colored symbols denoting a positive correlation with genes at various points in the pathway.

**Figure 7:**
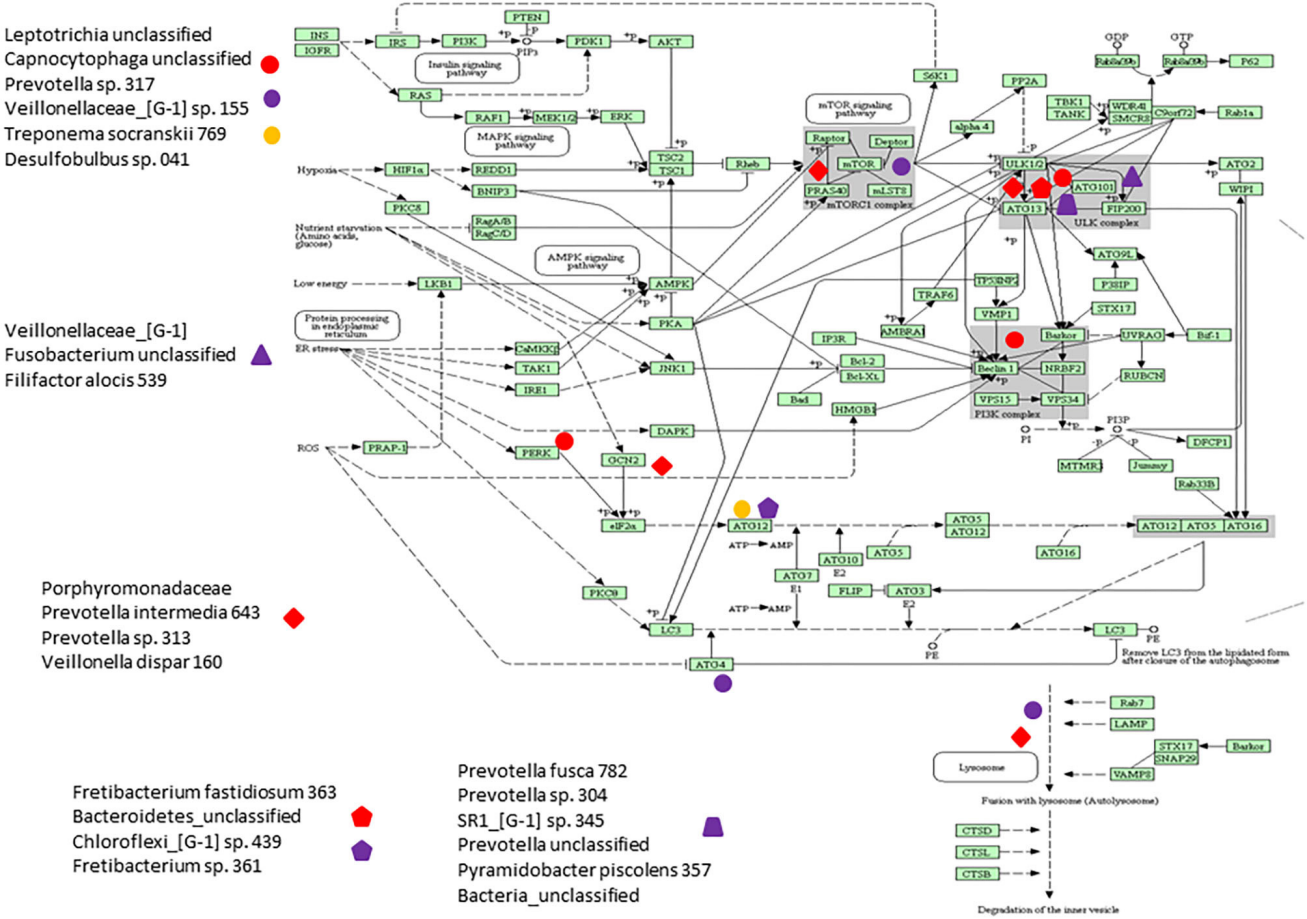
KEGG pathway graphic for autophagy. The bacterial complexes are identified with a relationship to autophagy genes at various points in the pathway with the red colored symbols denoting a negative correlation, the purple colored symbols denoting a positive correlation, and and the yellow colored symbol denotes a bacterial complex that is correlated both positively and negatively with genes in the ATG12 portions of the autophagy pathway.

Figure 6 presents a KEGG pathway schematic for apoptosis. The three bacterial complexes are included and genes correlated (positively or negatively) are highlighted in the pathway. As can be seen, the complexes not only appear to impact multiple points in the pathway, but these complexes overlap with certain pathway points and generally affect the genes in a similar direction. In particular, these correlated bacterial complexes are generally associated with positive relaionships (i.e., higher bacteria/higher mRNA; lower bacteria/lower mRNA) including both upstream signaling of the cellular pathway, as well as alterations in downstream molecules required to completing apoptosis outcomes. Figure 7 provides a similar schematic for the KEGG autophagy pathway. In this case the pathway steps that appear targeted by these bacterial complexes appear more limited with a predominance of bacterial complexes affecting the ULK initiation complex and three complexes negatively correlated with these genes two complexes negatively correlated with this initiation complex. Also, noted was that these bacterial complexes were also related to mTOR, ATG12, and LF/VD pathway steps with significant positive or negative correlations for gene regulation in the autophagy pathway.

## Discussion

Periodontal lesions develop from interactions of a dysbiotic oral microbiome and a juxtaposed dysregulated host response at sites of disease. There is clear evidence that the microbiome changes substantially from health to disease, with increases in overall burden, altered diversity, and emergence of various genera and species that are associated with and potentially causative of the host response dysregulation (50). However, the underlying mechanisms contributing to the breadth of host response changes including effects on resident cell functions, as well as alterations in infiltrating inflammatory and immune cells requires systems biology approaches and multi-omics analytics. These approaches are required to discern the earliest molecular stages of the disease process and transformations that occur for exacerbation or resolution of the lesion. As apoptosis and autophagy have been shown to be directly involved in the integrity of mucosal surfaces and regulation of localized inflammatory and immune responses, as well as likely contributing to the resolution of chronic inflammation, we sought to determine disease effects on these pathways and their relationship to microbiome conversion using a non-human primate model of ligature-induced periodontitis.

The results demonstrated specific alterations in the gingival transcriptome for both pro- and anti-apoptosis genes; however, the dominant changes with disease were increases in pro-apoptotic gene expression that generally occurred during disease initiation within 2 weeks of ligature placement and rapidly returned to baseline levels. Examination of changes in the autophagy-associated transcriptome, showed rather limited changes in the early events of the mTOR and ULK complexes. Interestingly, in the ULK complex both DRAM1 and DRAM2 were increased throughout disease, with both of these gene products important factors in regulating autophagy, and with increased combined levels also inducing apoptosis (51, 52). Some gene alterations in the PI3K complex were noted with increases during disease initiation. However, the most apparent alterations occurred in genes related to the ATG12 and lysosome fusion/vesicle degradation portions of the autophagy pathway. In this case, the ATG12 genes were generally increased with disease, while the LF/VD genes were decreased with disease initiation and progression. Thus, based upon this profile, it appears that a complete functional autophagy pathway to aid in host protection, would be predicted to be less effective in supporting recovery from cellular stress during periodontitis.

Beyond the clear differences in the host gingival response, changes in the oral microbiome also occur during ligature-induced periodontitis, in many ways similar to the differences that have been reported with human disease (43, 53). This study identified unique features of the microbiome in disease that significantly correlated with these changes in the gingival transcriptome. Specifically among the entirety of the 396 OTUs that were identified across the non-human primate samples (43), we identified a rather focused set of microorganisms that related to the differential expression of both apoptosis and autophagy genes. The identified microbial complexes included some members of the oral microbiome historically considered as pathogens (e.g., *Porphyromonadaceae, P. gingivalis, F. alocis*), while some of the complexes that appear to robustly correlate with these host pathways, are generally considered members of the commensal microbiota. The data from complexes AP-1 and AP-2 are striking, as one complex predominantly showed positive correlations with both anti- and pro- apoptotic genes and the other skewed toward negative correlations. Thus, the AP-1 complex could be stimulating signals that block apoptosis, whereas the AP-2 complex could be enhancing it. As importantly, it would be considered peculiar to have host-bacterial interactions that activate both pro- and anti-apoptotic signals simultaneously with potential pathologic consequences. With pro-apoptotic pathways up-regulated the final outcome would be expected to be increases in cell death competing with survival pathways that are also activated. These counteracting functions could be occurring in the same cells, which could lead to a substantial metabolic cost and loss of normal functions, or may reflect members of these bacterial complexes demonstrating differential signaling of pro- or anti-apoptotic processes in different cell types within the periodontium. The details of these options do present a limitation of the study design. That is, these gene expression patterns represent a holistic evaluation of the complex tissues, including epithelial cells, fibroblasts/connective tissue, vascular, and inflammatory/immune cells within the mucosal tissues in health and disease. While an argument can be made regarding the usefulness of determining individual cell type gene expression profiles in the periodontium, there nevertheless is inherent value in fundamentally detailing the alterations in these pathways comparing healthy to disease tissues. The findings describe altered apoptosis and autophagy gene expression in these tissues that is specifically associated with the disease process and generally return to a pattern reflecting healthy tissues with disease resolution. Importantly, the study presents seminal data regarding the relationship between specific microbes or microbial complexes in the oral microbiome and altered gene expression for these pathways. Also of interest was that there were clear similarities of members of the complexes that appear to impact both apoptosis and autophagy and show comparable correlation directions in these relationships. In particular, a complex comprised of *Leptotrichia, Veillonella, Capnocytophaga, Prevotella, T. socranskii*, and *Desulfobulbus* appeared very active in the relationship with both pathways. Moreover, the correlation of this complex with the host responses appeared to reflect multiple factors within the pathways and effects on genes whose products would be expected to disrupt a needed balance to maintain homeostasis. This outcome could reflect fundamental capabilities of these bacteria and their components to stimulate host cellular changes in the gingival environment and be related most directly to increases in their relative abundance in the disease microbiome and potentially organized into synergistic interactions within the biofilms.

Alternatively, these findings might reveal features related to the concept of dysbiosis and disease. More specifically, these outcomes could result from changes in metabolism and transcriptome of these normal commensal bacteria toward expression of genes that would be more deleterious to host cells and tissues, whereby they would actually contribute to the disease environment and clinical outcomes (14, 50, 54). As noted, simultaneous activation of pro-apoptotic pathways/genes and survival pathways might be considered pathologic at the individual cell level. This scenario could reflect an impaired cell death response that would contribute to persistent intracellular infection that can occur in periodontal tissues. Thus, some pathogens (e.g., *P. gingivalis, F. alocis*) and even commensals (e.g., *Veillonella*) could be involved in these impaired cell death responses by showing significant correlations with both pro- and anti-apoptotic gene expression. Additionally, the negative correlations with an increase in anti-apoptotic gene expression with decreased abundance of (OTUs) for specific bacteria, such as complex AP-2, could support a role for these particular bacterial species in inhibiting necessary apoptotic responses to maintain homeostasis. Further studies will be required to sort out these options, as well as determining the kinetics of changes occurring in the bacterial abundance that could contribute to a local environment that enhances tissue destructive events.

## Data Availability Statement

The datasets presented in this study can be found in online repositories. The names of the repository/repositories and accession number(s) can be found in the article/supplementary material.

## Ethics Statement

The animal study was reviewed and approved by Institutional Animal Care and Use Committee (IACUC) of the University of Puerto Rico.

## Author Contributions

JE and OG conceived of the studies, implemented the experiments, collected and analyzed the data, and prepared the manuscript. SK provided the analytics for the microbiome data and reviewing/revising the manuscript. EN provided an initial analysis of the host response data, an initial draft of the report, and reviewed the final manuscript. LO and JG provided the support for organizing the non-human primate studies and collection of samples and contributing to the methods section of the manuscript. All authors contributed to the article and approved the submitted version.

## Conflict of Interest

The authors declare that the research was conducted in the absence of any commercial or financial relationships that could be construed as a potential conflict of interest.
