# Supplementary material for: Oral Microbiome and Gingival Tissue Apoptosis and Autophagy Transcriptomics
Source: Front Immunol. 2020 Oct 19;11:585414. doi: 10.3389/fimmu.2020.585414 (PMC7604357; doi:10.3389/fimmu.2020.585414)
Supplement: Supplementary file 1 [file Data_Sheet_1.docx]

**Supplementary Table 1:** Microbiome correlations with apoptosis genes. Green denotes significantly negatively correlated and red denotes significantly positively correlated.

|  |  | **PIK3CG** | **CSF2RB** | **AKT3** | **BIRC3** | **PIK3CD** | **PRKACB** | **CFLAR** | **CD2** | **NOL3** | **IL1B** | **CASP3** | **TNFRS11B** | **APAF1** | **IL1RAP** |
| --- | --- | --- | --- | --- | --- | --- | --- | --- | --- | --- | --- | --- | --- | --- | --- |
| A. actinomycetemcomitans 531 | Otu017 | -0.0267 | -0.0339 | -0.0945 | 0.1256 | -0.0625 | -0.0081 | -0.0542 | -0.1315 | 0.1237 | -0.1032 | 0.0646 | -0.0484 | 0.1312 | -0.0468 |
| Aggregatibacter unclassified | Otu069 | 0.0186 | 0.1393 | 0.1639 | -0.0954 | 0.0954 | 0.0080 | -0.0537 | 0.2284 | -0.0741 | -0.1059 | -0.0292 | -0.0746 | -0.0256 | -0.1505 |
| Bacteria_unclassified | Otu003 | 0.1564 | -0.0649 | 0.0816 | -0.0680 | 0.0235 | 0.1853 | 0.0849 | 0.0865 | -0.2502 | -0.1536 | 0.0818 | -0.0334 | -0.0287 | -0.1484 |
| Bacteroidetes_unclassified | Otu020 | 0.0887 | -0.0039 | -0.0081 | -0.0040 | 0.0348 | 0.1781 | 0.0317 | 0.1220 | -0.1382 | -0.1842 | 0.0069 | -0.1184 | -0.0268 | -0.0866 |
| Capnocytophaga unclassified | Otu005 | -0.3468 | -0.3527 | -0.2825 | -0.2250 | -0.3075 | -0.1699 | -0.2186 | 0.0396 | 0.2477 | -0.1040 | -0.2981 | -0.1425 | -0.5173 | -0.2549 |
| Catonella morbi 165 | Otu014 | 0.0185 | 0.0068 | 0.0920 | -0.1512 | 0.0464 | -0.0291 | 0.0956 | -0.0568 | -0.0197 | 0.0611 | -0.0489 | 0.0119 | 0.0404 | 0.1140 |
| Chloroflexi_[G-1] sp. 439 | Otu030 | 0.1619 | 0.1041 | 0.0887 | 0.1222 | 0.0820 | 0.2318 | 0.1749 | 0.2288 | -0.3622 | -0.1307 | 0.0869 | -0.1900 | -0.0373 | -0.0447 |
| Desulfobulbus sp. 041 | Otu084 | -0.2749 | -0.2682 | -0.2486 | -0.1480 | -0.1714 | -0.1057 | -0.2308 | -0.0282 | 0.0440 | -0.1377 | -0.1109 | -0.1265 | -0.3431 | -0.1066 |
| Eubacterium infirmum 105 | Otu037 | 0.1587 | -0.0893 | 0.0116 | 0.0014 | 0.0169 | -0.1180 | 0.0511 | 0.0452 | -0.0598 | -0.1512 | 0.0310 | -0.1107 | 0.0365 | -0.1217 |
| Filifactor alocis 539 | Otu063 | 0.3517 | 0.3393 | 0.2781 | 0.1961 | 0.2861 | 0.4123 | 0.3387 | -0.0498 | -0.5210 | 0.0593 | 0.4402 | 0.1152 | 0.4535 | 0.2801 |
| Fretibacterium fastidiosum 363 | Otu027 | -0.0669 | -0.1024 | 0.0379 | 0.0616 | 0.0075 | 0.1186 | -0.1017 | 0.1192 | -0.0072 | -0.1241 | -0.0346 | -0.0710 | 0.0705 | -0.0246 |
| Fretibacterium sp. 361 | Otu009 | 0.0701 | 0.0332 | 0.1964 | 0.1180 | 0.2519 | 0.2207 | 0.0598 | 0.1873 | -0.2256 | -0.0293 | 0.0998 | 0.0263 | 0.1288 | 0.0358 |
| Fretibacterium unclassified | Otu012 | -0.2275 | -0.1284 | -0.1898 | -0.1092 | -0.1832 | -0.1539 | -0.1683 | -0.1622 | 0.2069 | 0.1013 | -0.0389 | 0.0794 | -0.2207 | 0.0684 |
| Fusobacterium sp. 203 | Otu060 | -0.2329 | -0.1150 | -0.3745 | -0.2906 | -0.2157 | -0.2217 | -0.2134 | -0.0070 | 0.2352 | 0.0318 | -0.3332 | -0.1608 | -0.2806 | -0.1279 |
| Fusobacterium unclassified | Otu001 | 0.0056 | 0.2406 | 0.2008 | 0.1065 | 0.3130 | -0.0120 | 0.0696 | -0.1838 | -0.0165 | 0.3001 | 0.1994 | 0.1999 | 0.2674 | 0.3796 |
| Gemella morbillorum 046 | Otu045 | 0.0326 | 0.2196 | 0.1144 | 0.0833 | 0.2582 | -0.0632 | 0.0738 | -0.1091 | 0.0672 | 0.2893 | 0.0680 | 0.3397 | 0.1255 | 0.3000 |
| Haemophilus sp. 035 | Otu093 | -0.1082 | 0.0548 | 0.0273 | -0.0483 | 0.0298 | -0.1480 | -0.1013 | -0.1924 | 0.1505 | -0.0340 | -0.0111 | 0.0807 | 0.0354 | 0.0868 |
| Leptotrichia sp. 223 | Otu032 | -0.2261 | -0.3192 | -0.2041 | -0.1879 | -0.2790 | -0.1621 | -0.1717 | -0.0078 | 0.1205 | -0.1212 | -0.2501 | -0.1184 | -0.2893 | -0.1450 |
| Leptotrichia unclassified | Otu053 | -0.3296 | -0.3763 | -0.3063 | -0.2780 | -0.3327 | -0.1839 | -0.2019 | -0.1987 | 0.2378 | -0.0160 | -0.2296 | -0.0540 | -0.4323 | -0.0792 |
| Megasphaera micronuciformis 122 | Otu092 | -0.1533 | -0.0554 | -0.2270 | -0.0781 | -0.2457 | -0.3122 | -0.1826 | -0.0875 | 0.2927 | 0.0455 | -0.2316 | -0.1352 | -0.1521 | -0.1233 |
| Moraxella catarrhalis 833 | Otu170 | -0.1969 | -0.1631 | -0.1035 | -0.0210 | -0.1101 | -0.2687 | -0.1024 | -0.0653 | 0.1346 | -0.0025 | -0.1996 | 0.0045 | -0.1675 | -0.0600 |
| Neisseria oralis 014 | Otu074 | -0.1542 | 0.0910 | -0.0078 | -0.2361 | -0.1770 | -0.0408 | -0.1227 | -0.0647 | 0.0765 | 0.0940 | -0.0787 | -0.1633 | -0.1153 | 0.0237 |
| Pasteurellaceae_unclassified | Otu019 | 0.0836 | 0.1430 | 0.2179 | 0.1039 | 0.2266 | 0.1990 | 0.0223 | 0.1214 | -0.1961 | 0.0673 | 0.0964 | 0.1358 | 0.1556 | 0.2018 |
| Peptostreptococcaceae_[XI]_unclassified | Otu046 | -0.0164 | -0.1041 | -0.0024 | -0.0681 | 0.0998 | 0.1386 | -0.0263 | 0.1083 | -0.1644 | -0.1340 | -0.0088 | -0.1743 | -0.0993 | -0.1003 |
| Peptostreptococcaceae_[XIII]_unclassified | Otu067 | 0.1261 | 0.1212 | 0.2124 | 0.0427 | 0.1357 | 0.2488 | 0.0769 | -0.0049 | -0.2716 | -0.0750 | 0.2077 | 0.0997 | 0.2496 | 0.1432 |
| Porphyromonadaceae | Otu039 | 0.3406 | 0.5258 | 0.3441 | 0.2890 | 0.4744 | 0.2556 | 0.3161 | 0.0959 | -0.3548 | 0.5503 | 0.2937 | 0.4423 | 0.3531 | 0.5011 |
| Porphyromonas endodontalis 273 | Otu033 | 0.0986 | 0.0638 | 0.1608 | 0.0225 | 0.1540 | 0.1972 | 0.1196 | -0.1477 | -0.2021 | -0.0387 | 0.2038 | -0.1177 | 0.3090 | 0.1511 |
| Porphyromonas gingivalis 619 | Otu021 | -0.0109 | 0.0236 | 0.2514 | 0.0377 | 0.2548 | 0.1976 | 0.1204 | -0.1359 | -0.1966 | 0.1480 | 0.2017 | 0.1969 | 0.3976 | 0.3289 |
| Porphyromonas sp. 279 | Otu022 | -0.0693 | 0.1127 | 0.0525 | 0.0099 | 0.1470 | -0.1178 | -0.0286 | -0.1062 | 0.1539 | 0.1046 | -0.0152 | 0.1460 | 0.0501 | 0.1651 |
| Prevotella denticola 291 | Otu116 | -0.1774 | -0.0508 | -0.2515 | -0.0907 | -0.2554 | -0.3108 | -0.1959 | -0.0758 | 0.2952 | 0.0587 | -0.2604 | -0.1583 | -0.1662 | -0.0944 |
| Prevotella enoeca 600 | Otu023 | -0.2273 | -0.2023 | -0.0377 | -0.1159 | -0.0414 | -0.1613 | -0.1116 | -0.0337 | 0.0652 | 0.0239 | -0.1399 | -0.2139 | -0.0177 | 0.0177 |
| Prevotella fusca 782 | Otu082 | 0.3112 | 0.1667 | 0.0576 | -0.0729 | 0.1189 | -0.0220 | 0.3070 | -0.0070 | -0.0716 | 0.0454 | 0.1686 | 0.0557 | 0.1258 | -0.0493 |
| Prevotella intermedia 643 | Otu062 | 0.2383 | 0.2172 | 0.1761 | 0.3306 | 0.0715 | 0.3679 | 0.1698 | 0.1086 | -0.2392 | -0.0684 | 0.3318 | 0.0930 | 0.2698 | 0.0749 |
| Prevotella sp. 304 | Otu056 | 0.1713 | 0.0681 | 0.0531 | 0.0150 | 0.1706 | 0.0821 | 0.3077 | -0.1309 | -0.2013 | 0.0571 | 0.1182 | -0.1323 | 0.3769 | 0.1740 |
| Prevotella sp. 311 | Otu040 | -0.1680 | -0.0822 | -0.2513 | -0.0637 | -0.2943 | -0.2651 | -0.2382 | -0.1115 | 0.2593 | -0.0285 | -0.1770 | -0.1116 | -0.0660 | -0.0660 |
| Prevotella sp. 313 | Otu059 | 0.2271 | 0.0796 | 0.0897 | 0.1279 | 0.0944 | -0.0194 | 0.0598 | -0.1146 | -0.0939 | -0.0275 | 0.2225 | 0.0910 | 0.1302 | 0.0059 |
| Prevotella sp. 317 | Otu004 | -0.2668 | -0.1846 | -0.2011 | -0.2756 | -0.2874 | -0.2258 | -0.1622 | 0.1777 | 0.1747 | -0.0809 | -0.4070 | -0.1847 | -0.2776 | -0.2088 |
| Prevotella sp. 443 | Otu102 | 0.2176 | 0.2869 | 0.0103 | 0.0167 | -0.0276 | 0.1035 | 0.0857 | 0.2462 | -0.0152 | -0.2072 | 0.0320 | -0.1339 | -0.0067 | -0.3111 |
| Prevotella sp. 526 | Otu013 | 0.0538 | 0.0007 | 0.0653 | 0.0071 | -0.0113 | 0.0347 | -0.0573 | 0.1451 | 0.0434 | -0.1956 | -0.0600 | -0.1091 | 0.0178 | -0.1617 |
| Prevotella sp. 820 | Otu041 | 0.0484 | 0.0133 | -0.0346 | 0.0394 | -0.1312 | 0.0483 | 0.0224 | -0.1287 | -0.0528 | -0.1690 | 0.1385 | 0.0222 | 0.0964 | -0.1100 |
| Prevotella unclassified | Otu008 | 0.1102 | -0.0733 | -0.0174 | 0.0735 | -0.0536 | -0.0861 | 0.1497 | 0.1400 | -0.0275 | -0.0249 | -0.0575 | -0.1221 | 0.0227 | -0.0844 |
| Pyramidobacter piscolens 357 | Otu050 | 0.1392 | -0.0846 | -0.0288 | -0.0848 | -0.0191 | 0.1638 | 0.0832 | -0.1147 | -0.1512 | -0.1027 | 0.1318 | 0.0337 | 0.0162 | -0.0447 |
| Selenomonas sputigena 151 | Otu011 | -0.0621 | -0.1380 | -0.1419 | -0.2782 | -0.2858 | -0.0096 | -0.0696 | 0.0764 | 0.1259 | -0.1451 | -0.2267 | -0.0474 | -0.2144 | -0.2455 |
| Selenomonas unclassified | Otu073 | -0.2500 | -0.2916 | -0.2889 | -0.2218 | -0.2820 | -0.2533 | -0.1989 | -0.1676 | 0.1656 | -0.0377 | -0.1969 | -0.1351 | -0.2981 | -0.1253 |
| SR1_[G-1] sp. 345 | Otu028 | 0.2265 | 0.0375 | 0.0933 | 0.1047 | 0.0535 | 0.2088 | 0.1751 | 0.0128 | -0.1754 | -0.1871 | 0.1536 | -0.0582 | 0.2256 | -0.0346 |
| Streptococcus | Otu007 | 0.1388 | 0.0608 | 0.0201 | -0.0107 | -0.0098 | -0.0259 | 0.1374 | 0.1304 | -0.0237 | -0.0783 | 0.0243 | -0.1594 | -0.0485 | -0.1342 |
| Streptococcus parasanguinis II 411 | Otu064 | -0.0041 | 0.1274 | -0.0354 | -0.0209 | 0.0064 | -0.1775 | -0.0674 | -0.0749 | 0.1008 | 0.0583 | 0.0757 | 0.0550 | -0.1480 | -0.0134 |
| Streptococcus sp. 058 | Otu024 | 0.1349 | 0.1372 | 0.0844 | -0.0095 | 0.0810 | -0.0298 | 0.0511 | -0.1171 | -0.0375 | -0.0158 | 0.1447 | 0.0365 | 0.0922 | 0.0595 |
| Treponema denticola 584 | Otu018 | -0.1205 | -0.0774 | -0.1254 | -0.0563 | -0.1259 | -0.0654 | 0.0271 | -0.0587 | 0.0752 | -0.1041 | -0.0561 | -0.1410 | -0.0571 | -0.1416 |
| Treponema maltophilum 664 | Otu026 | 0.0813 | 0.0791 | 0.1682 | 0.1400 | 0.3238 | 0.2694 | 0.1175 | 0.2169 | -0.0722 | 0.2142 | 0.0793 | 0.1848 | 0.1007 | 0.1552 |
| Treponema socranskii 769 | Otu015 | -0.3576 | -0.1990 | -0.2592 | -0.0944 | -0.1013 | -0.1914 | -0.1177 | -0.0513 | 0.2140 | 0.2115 | -0.2346 | 0.1808 | -0.3550 | 0.0430 |
| Treponema sp. 246 | Otu104 | -0.0985 | -0.0790 | 0.0780 | 0.2624 | 0.0763 | 0.0577 | -0.0009 | 0.1218 | 0.0016 | -0.0348 | 0.0454 | 0.0747 | 0.1057 | 0.0702 |
| Treponema unclassified | Otu002 | 0.0680 | 0.0858 | 0.1061 | 0.1426 | -0.0113 | 0.1776 | 0.1568 | -0.0624 | -0.2169 | -0.1084 | 0.2057 | 0.0383 | 0.2120 | 0.1051 |
| Veillonella dispar 160 | Otu058 | 0.2653 | 0.1352 | 0.0343 | 0.2076 | 0.0967 | 0.0001 | 0.0149 | -0.0398 | -0.0389 | 0.0008 | 0.2639 | 0.1111 | 0.0137 | 0.0296 |
| Veillonella parvula 161 | Otu010 | 0.0219 | -0.0887 | -0.0175 | 0.1158 | -0.0720 | -0.0090 | -0.0615 | 0.1137 | 0.0551 | -0.0981 | -0.0372 | -0.0722 | -0.1007 | -0.1724 |
| Veillonella unclassified | Otu006 | 0.2273 | 0.0565 | 0.1636 | 0.0609 | 0.0319 | 0.1622 | 0.1413 | 0.0916 | -0.2050 | -0.0076 | 0.1886 | 0.0757 | 0.0425 | -0.0441 |
| Veillonellaceae_[G-1] | Otu025 | 0.4066 | 0.5284 | 0.3188 | 0.2084 | 0.5687 | 0.2546 | 0.3403 | 0.1512 | -0.2408 | 0.4816 | 0.3068 | 0.4235 | 0.4786 | 0.4534 |
| Veillonellaceae_[G-1] sp. 155 | Otu048 | -0.3863 | -0.4296 | -0.3187 | -0.2116 | -0.3618 | -0.2526 | -0.2027 | -0.3035 | 0.2592 | -0.0120 | -0.2440 | 0.0533 | -0.3893 | -0.0376 |
|  |  | **CASP7** | **DAPK1** | **CASP10** | **CASP8** | **CASP1** | **IRAK3** | **PRKAR2B** | **ATM** | **BID** | **IL1R1** | **TRAF3** | **IL1A** | **ENDOD1** |  |
| A. actinomycetemcomitans 531 | Otu017 | -0.0426 | -0.1380 | -0.0458 | -0.0864 | -0.0265 | 0.0940 | -0.1346 | -0.0437 | 0.0243 | -0.0897 | 0.1367 | -0.1078 | 0.0610 |  |
| Aggregatibacter unclassified | Otu069 | 0.0630 | 0.1650 | 0.2426 | 0.0827 | -0.0169 | 0.0109 | -0.0155 | 0.0717 | -0.0094 | 0.1588 | 0.0204 | 0.2080 | -0.0167 |  |
| Bacteria_unclassified | Otu003 | 0.0144 | -0.0503 | 0.0018 | 0.0680 | -0.2702 | -0.0059 | 0.2045 | 0.1724 | 0.1305 | -0.1379 | 0.1391 | -0.0501 | -0.0459 |  |
| Bacteroidetes_unclassified | Otu020 | -0.1306 | -0.0616 | -0.0854 | -0.0001 | -0.3038 | 0.0239 | -0.0223 | 0.1575 | 0.0274 | -0.1621 | 0.0747 | -0.0532 | -0.1916 |  |
| Capnocytophaga unclassified | Otu005 | -0.1118 | -0.3155 | -0.2928 | -0.2491 | -0.3633 | -0.3687 | 0.0590 | -0.2346 | -0.3526 | -0.2180 | -0.4334 | 0.1430 | 0.3401 |  |
| Catonella morbi 165 | Otu014 | 0.1360 | 0.1384 | 0.0991 | -0.0337 | -0.0146 | 0.0747 | -0.0412 | -0.0202 | 0.0159 | 0.2932 | -0.1586 | -0.1606 | -0.0699 |  |
| Chloroflexi_[G-1] sp. 439 | Otu030 | 0.0563 | 0.0330 | 0.0341 | 0.1453 | -0.0502 | 0.0543 | 0.1336 | 0.2384 | 0.1565 | -0.0700 | 0.2192 | -0.0460 | -0.2506 |  |
| Desulfobulbus sp. 041 | Otu084 | -0.1171 | -0.2750 | -0.3454 | -0.2275 | -0.0142 | -0.1931 | -0.0952 | -0.2120 | -0.2260 | -0.2746 | -0.1111 | -0.1566 | 0.2162 |  |
| Eubacterium infirmum 105 | Otu037 | -0.1289 | 0.0748 | 0.1163 | -0.0449 | 0.2118 | 0.0109 | -0.1875 | 0.1139 | -0.1062 | -0.0148 | 0.0627 | -0.3040 | -0.0262 |  |
| Filifactor alocis 539 | Otu063 | 0.4088 | 0.2606 | 0.3272 | 0.3006 | 0.1372 | 0.4242 | 0.2112 | 0.3441 | 0.3810 | 0.2585 | 0.4159 | -0.1053 | -0.5145 |  |
| Fretibacterium fastidiosum 363 | Otu027 | -0.1156 | -0.0220 | -0.1291 | -0.0242 | 0.1029 | 0.1761 | -0.0301 | -0.0108 | 0.0284 | -0.0394 | 0.0962 | -0.2432 | 0.0113 |  |
| Fretibacterium sp. 361 | Otu009 | 0.0295 | 0.1342 | -0.0171 | 0.1271 | 0.0136 | 0.2448 | 0.0378 | 0.0949 | 0.1775 | 0.0770 | 0.1940 | -0.2175 | -0.1890 |  |
| Fretibacterium unclassified | Otu012 | 0.0362 | -0.1393 | -0.2282 | -0.1438 | 0.1858 | -0.2777 | -0.0265 | -0.1977 | -0.2194 | -0.1713 | -0.1656 | 0.1043 | 0.2763 |  |
| Fusobacterium sp. 203 | Otu060 | -0.2269 | -0.2387 | -0.2828 | -0.2424 | -0.0901 | -0.3413 | -0.1366 | -0.2420 | -0.2241 | -0.2421 | -0.3177 | 0.2437 | 0.2788 |  |
| Fusobacterium unclassified | Otu001 | 0.2479 | 0.3156 | 0.1140 | 0.0889 | 0.3043 | 0.1974 | 0.0834 | -0.1283 | 0.2841 | 0.2818 | 0.1557 | -0.0001 | -0.2210 |  |
| Gemella morbillorum 046 | Otu045 | 0.1748 | 0.1777 | 0.1119 | 0.0499 | -0.0097 | 0.0814 | 0.0024 | -0.1541 | 0.2543 | 0.2211 | 0.0701 | 0.1377 | -0.1693 |  |
| Haemophilus sp. 035 | Otu093 | 0.0195 | 0.0244 | 0.0341 | -0.1430 | -0.1190 | -0.0326 | -0.0459 | -0.1906 | 0.0487 | 0.0601 | -0.0764 | 0.1120 | -0.0792 |  |
| Leptotrichia sp. 223 | Otu032 | -0.1056 | -0.2437 | -0.2717 | -0.1837 | -0.1481 | -0.0990 | -0.2125 | -0.0835 | -0.3901 | -0.0726 | -0.3451 | -0.1028 | 0.2894 |  |
| Leptotrichia unclassified | Otu053 | -0.0005 | -0.3010 | -0.3359 | -0.2855 | -0.1705 | -0.3047 | 0.0283 | -0.2703 | -0.3174 | -0.1623 | -0.4313 | -0.0556 | 0.3518 |  |
| Megasphaera micronuciformis 122 | Otu092 | -0.1968 | -0.1562 | -0.2451 | -0.1542 | 0.0332 | -0.1792 | -0.1954 | -0.1799 | -0.1423 | -0.0700 | -0.1883 | 0.0206 | 0.3348 |  |
| Moraxella catarrhalis 833 | Otu170 | -0.1758 | -0.0817 | -0.1809 | -0.2617 | 0.0213 | -0.0435 | -0.2049 | -0.2295 | -0.1555 | 0.0266 | -0.1592 | -0.0400 | 0.1791 |  |
| Neisseria oralis 014 | Otu074 | 0.0505 | -0.0069 | -0.0348 | -0.0801 | -0.0554 | -0.0899 | 0.0892 | -0.1340 | -0.0238 | -0.0918 | -0.2712 | 0.1846 | 0.0495 |  |
| Pasteurellaceae_unclassified | Otu019 | 0.1401 | 0.1467 | 0.0174 | 0.0634 | -0.0289 | 0.0851 | 0.0580 | 0.1115 | 0.2810 | 0.1168 | 0.0132 | -0.0066 | -0.1986 |  |
| Peptostreptococcaceae_[XI]_unclassified | Otu046 | -0.0546 | 0.0273 | -0.1849 | -0.0153 | -0.2284 | -0.0908 | 0.0241 | 0.0945 | -0.0213 | -0.2185 | -0.0446 | -0.0863 | -0.1361 |  |
| Peptostreptococcaceae_[XIII]_unclassified | Otu067 | 0.2842 | 0.1253 | 0.1056 | 0.1265 | 0.0886 | 0.3173 | 0.2300 | 0.1228 | 0.2657 | 0.1679 | 0.2280 | -0.2012 | -0.2705 |  |
| Porphyromonadaceae | Otu039 | 0.4707 | 0.3933 | 0.2237 | 0.3808 | 0.3010 | 0.2692 | 0.0973 | 0.1347 | 0.6592 | 0.4176 | 0.3181 | 0.0583 | -0.3387 |  |
| Porphyromonas endodontalis 273 | Otu033 | 0.2786 | 0.1921 | 0.1863 | 0.0816 | 0.2518 | 0.2932 | -0.0633 | 0.1219 | 0.1926 | 0.2502 | 0.1792 | -0.2532 | -0.1422 |  |
| Porphyromonas gingivalis 619 | Otu021 | 0.2483 | 0.3322 | 0.1449 | 0.1416 | 0.3996 | 0.4087 | 0.1897 | 0.0640 | 0.1971 | 0.3813 | 0.1629 | -0.2796 | -0.1907 |  |
| Porphyromonas sp. 279 | Otu022 | 0.0839 | 0.0736 | 0.0286 | -0.0712 | -0.0949 | 0.0210 | -0.0720 | -0.1684 | 0.1182 | 0.1148 | -0.0280 | 0.1209 | -0.1123 |  |
| Prevotella denticola 291 | Otu116 | -0.1927 | -0.1594 | -0.2639 | -0.1767 | 0.0545 | -0.1739 | -0.2242 | -0.1967 | -0.1584 | -0.0651 | -0.2115 | 0.0120 | 0.3326 |  |
| Prevotella enoeca 600 | Otu023 | -0.1288 | 0.0756 | -0.1810 | -0.0750 | 0.2564 | 0.0420 | -0.2093 | -0.0603 | -0.3188 | 0.1437 | -0.0805 | -0.2262 | 0.1812 |  |
| Prevotella fusca 782 | Otu082 | -0.1025 | 0.0989 | 0.1568 | 0.1359 | 0.0007 | -0.0698 | -0.0168 | 0.1971 | 0.0415 | 0.0786 | 0.1380 | -0.0530 | -0.1524 |  |
| Prevotella intermedia 643 | Otu062 | 0.2891 | 0.0766 | 0.2160 | 0.1828 | 0.0569 | 0.0766 | 0.1385 | 0.2823 | 0.3304 | 0.0302 | 0.2101 | 0.0616 | -0.2223 |  |
| Prevotella sp. 304 | Otu056 | -0.0178 | 0.2890 | 0.2579 | 0.1725 | 0.1546 | 0.3201 | -0.0613 | 0.2234 | -0.0094 | 0.3130 | 0.1868 | -0.3495 | -0.2296 |  |
| Prevotella sp. 311 | Otu040 | -0.2255 | -0.1769 | -0.1956 | -0.1838 | 0.0349 | -0.1747 | -0.2257 | -0.1259 | -0.1415 | -0.1214 | -0.2009 | -0.0528 | 0.3332 |  |
| Prevotella sp. 313 | Otu059 | 0.0022 | 0.0800 | 0.2034 | 0.0465 | 0.2774 | 0.0516 | 0.0126 | 0.1009 | 0.0528 | 0.0308 | 0.0190 | -0.0358 | -0.1851 |  |
| Prevotella sp. 317 | Otu004 | -0.2957 | -0.1742 | -0.1988 | -0.2029 | -0.1188 | -0.2155 | -0.1577 | -0.1174 | -0.3988 | -0.0478 | -0.3973 | 0.1626 | 0.2598 |  |
| Prevotella sp. 443 | Otu102 | -0.0810 | -0.0303 | 0.2274 | 0.1360 | -0.1460 | -0.0447 | 0.0524 | 0.1023 | 0.0935 | -0.2125 | 0.1233 | 0.3946 | -0.1673 |  |
| Prevotella sp. 526 | Otu013 | -0.0967 | 0.0005 | 0.0357 | -0.0838 | 0.1068 | -0.0355 | -0.1290 | 0.0134 | 0.0868 | -0.0962 | 0.1293 | -0.0473 | -0.0688 |  |
| Prevotella sp. 820 | Otu041 | 0.0237 | -0.0791 | 0.0433 | -0.0208 | -0.0337 | 0.0717 | 0.0248 | 0.0555 | 0.0411 | -0.0378 | 0.0546 | 0.0642 | 0.0598 |  |
| Prevotella unclassified | Otu008 | -0.0433 | 0.0199 | 0.2071 | 0.1627 | -0.0333 | 0.1075 | -0.1360 | 0.2035 | -0.2735 | 0.0781 | 0.1219 | -0.1335 | 0.1313 |  |
| Pyramidobacter piscolens 357 | Otu050 | -0.0200 | -0.1411 | -0.0114 | -0.0727 | -0.2372 | -0.0725 | 0.1491 | 0.1167 | 0.1122 | -0.1162 | 0.0667 | -0.1138 | -0.0376 |  |
| Selenomonas sputigena 151 | Otu011 | -0.1822 | -0.1869 | -0.0728 | -0.1131 | -0.2233 | -0.3300 | 0.1682 | -0.0549 | -0.1971 | -0.1941 | -0.2404 | 0.3061 | 0.2125 |  |
| Selenomonas unclassified | Otu073 | -0.0344 | -0.2060 | -0.2557 | -0.1357 | -0.0400 | -0.1781 | -0.2286 | -0.1776 | -0.3301 | -0.2108 | -0.1959 | 0.0643 | 0.4617 |  |
| SR1_[G-1] sp. 345 | Otu028 | 0.0948 | 0.0418 | 0.2109 | 0.1202 | 0.0351 | 0.1751 | -0.0394 | 0.2515 | 0.1486 | -0.0451 | 0.3424 | -0.1621 | -0.1623 |  |
| Streptococcus | Otu007 | -0.0003 | 0.0193 | 0.2385 | 0.1608 | -0.2079 | -0.0328 | -0.0266 | 0.1263 | -0.1027 | 0.0127 | 0.0201 | 0.0295 | -0.0230 |  |
| Streptococcus parasanguinis II 411 | Otu064 | 0.0216 | -0.0012 | 0.1421 | -0.0974 | 0.1019 | -0.1466 | -0.0742 | -0.1371 | -0.0647 | -0.0484 | -0.1355 | 0.2751 | 0.0158 |  |
| Streptococcus sp. 058 | Otu024 | 0.1624 | 0.1483 | 0.3094 | 0.0627 | -0.0764 | 0.1207 | 0.0549 | 0.0237 | 0.0339 | 0.0669 | 0.0778 | 0.0944 | -0.1494 |  |
| Treponema denticola 584 | Otu018 | -0.1222 | -0.0433 | -0.0609 | -0.0258 | 0.0034 | 0.0775 | 0.0015 | -0.1053 | -0.1200 | -0.1261 | 0.0488 | -0.0836 | -0.0094 |  |
| Treponema maltophilum 664 | Otu026 | 0.1409 | 0.1998 | -0.0298 | 0.1818 | 0.0888 | 0.1105 | 0.0816 | 0.1004 | 0.1988 | 0.0722 | 0.1316 | -0.0350 | -0.1113 |  |
| Treponema socranskii 769 | Otu015 | 0.0036 | -0.1862 | -0.2929 | -0.1729 | -0.0442 | -0.1776 | -0.1082 | -0.3482 | -0.1425 | -0.0454 | -0.3324 | 0.1392 | 0.3064 |  |
| Treponema sp. 246 | Otu104 | -0.0760 | 0.0247 | -0.1086 | 0.0825 | 0.1617 | 0.1221 | 0.0744 | 0.0219 | 0.0236 | -0.0246 | 0.1094 | -0.1405 | -0.0754 |  |
| Treponema unclassified | Otu002 | 0.0533 | 0.0468 | 0.1179 | 0.1429 | 0.0220 | 0.2889 | 0.1438 | 0.1631 | 0.0322 | -0.0282 | 0.2820 | -0.0476 | -0.1933 |  |
| Veillonella dispar 160 | Otu058 | -0.0427 | -0.0568 | 0.1071 | -0.0253 | 0.2771 | -0.0960 | -0.0276 | 0.1078 | 0.0433 | -0.1168 | -0.0337 | 0.0182 | -0.2434 |  |
| Veillonella parvula 161 | Otu010 | -0.1399 | -0.1708 | 0.0115 | 0.0394 | -0.1146 | -0.0817 | 0.0425 | 0.0948 | -0.1434 | -0.1037 | -0.0574 | 0.0414 | 0.0519 |  |
| Veillonella unclassified | Otu006 | 0.1991 | 0.1401 | 0.3139 | 0.2389 | -0.0780 | 0.1195 | 0.2140 | 0.2410 | -0.0379 | 0.0667 | 0.1507 | 0.0523 | -0.0477 |  |
| Veillonellaceae_[G-1] | Otu025 | 0.3255 | 0.4053 | 0.3299 | 0.4086 | 0.0894 | 0.4217 | -0.1607 | 0.2177 | 0.5379 | 0.3776 | 0.4695 | -0.0249 | -0.3686 |  |
| Veillonellaceae_[G-1] sp. 155 | Otu048 | -0.1158 | -0.2747 | -0.3585 | -0.3439 | -0.0919 | -0.2628 | 0.1000 | -0.3525 | -0.2538 | -0.1789 | -0.3934 | -0.1743 | 0.3998 |  |

**Supplementary Table 2:** Microbiome correlations with autophagy genes. Green denotes significantly negatively correlated and red denotes significantly positively correlated.

|  |  | **EIF4G1** | **LAMTOR3** | **ATG101** | **DRAM1** | **DRAM2** | **SNX4** | **ATG14** | **DAPK1** | **IGF1** | **PIK3CG** | **INPPL1** |
| --- | --- | --- | --- | --- | --- | --- | --- | --- | --- | --- | --- | --- |
| A. actinomycetemcomitans 531 | Otu017 | -0.0209 | 0.0160 | 0.0585 | -0.0011 | -0.0435 | 0.0357 | -0.0039 | -0.1156 | -0.0965 | -0.0760 | -0.0309 |
| Aggregatibacter unclassified | Otu069 | -0.0600 | -0.0546 | 0.0352 | 0.0726 | 0.0719 | -0.1011 | 0.0155 | 0.0608 | 0.0570 | 0.0155 | 0.1220 |
| Bacteria_unclassified | Otu003 | 0.1164 | 0.1468 | 0.1325 | -0.0650 | 0.1249 | 0.2793 | -0.1736 | -0.0135 | 0.3170 | 0.0696 | 0.0942 |
| Bacteroidetes_unclassified | Otu020 | 0.0859 | -0.1299 | 0.2193 | -0.0506 | -0.1374 | -0.0680 | -0.0580 | -0.0458 | 0.2264 | 0.0273 | 0.1163 |
| Capnocytophaga unclassified | Otu015 | 0.2752 | 0.0752 | -0.0691 | -0.1216 | -0.2910 | -0.1147 | 0.1512 | -0.2313 | -0.3578 | -0.3279 | -0.3184 |
| Catonella morbi 165 | Otu014 | 0.0258 | -0.1448 | 0.1668 | 0.1085 | -0.0612 | 0.0900 | 0.0110 | 0.0959 | -0.1691 | -0.0032 | 0.0420 |
| Chloroflexi_[G-1] sp. 439 | Otu030 | -0.0873 | -0.1663 | 0.1964 | -0.0420 | 0.0367 | 0.0068 | -0.1432 | 0.1039 | 0.3383 | 0.0884 | 0.3332 |
| Desulfobulbus sp. 041 | Otu084 | 0.1248 | -0.1412 | 0.3136 | -0.2286 | -0.3222 | -0.2366 | 0.2067 | -0.2468 | 0.0087 | -0.3057 | -0.0657 |
| Eubacterium infirmum 105 | Otu037 | -0.2048 | -0.1416 | 0.0901 | -0.1272 | 0.0182 | 0.1287 | -0.0344 | 0.0562 | 0.0642 | 0.0788 | -0.1053 |
| Filifactor alocis 539 | Otu063 | -0.1614 | -0.0211 | 0.2424 | 0.2822 | 0.3284 | 0.1387 | -0.2701 | 0.2571 | 0.4998 | 0.2234 | 0.3798 |
| Fretibacterium fastidiosum 363 | Otu027 | -0.0902 | -0.2086 | 0.3077 | 0.0517 | -0.1090 | -0.1569 | -0.0877 | -0.0164 | 0.1379 | -0.1146 | 0.0581 |
| Fretibacterium sp. 361 | Otu009 | -0.0077 | -0.2469 | 0.4415 | 0.1139 | -0.0252 | -0.1902 | -0.1544 | 0.0919 | 0.3308 | -0.0184 | 0.2168 |
| Fretibacterium unclassified | Otu012 | 0.0745 | 0.0615 | -0.1889 | -0.1159 | -0.2070 | -0.1284 | 0.3094 | -0.0901 | -0.3131 | -0.2032 | -0.2005 |
| Fusobacterium sp. 203 | Otu032 | 0.1771 | 0.0126 | 0.1025 | -0.1681 | -0.1342 | 0.1014 | 0.2129 | -0.2244 | -0.2571 | -0.2058 | -0.1424 |
| Fusobacterium unclassified | Otu001 | -0.1877 | -0.1179 | 0.1618 | 0.3137 | 0.0618 | -0.2723 | -0.1722 | 0.2776 | -0.1439 | -0.0217 | 0.0845 |
| Gemella morbillorum 046 | Otu045 | 0.0756 | 0.0867 | 0.1171 | 0.2698 | 0.0083 | -0.0500 | -0.1446 | 0.1235 | -0.2419 | 0.0692 | -0.0311 |
| Haemophilus sp. 035 | Otu093 | 0.0142 | 0.1142 | 0.0843 | 0.1362 | -0.0482 | -0.1123 | -0.0323 | 0.0147 | -0.2282 | -0.0841 | 0.0262 |
| Leptotrichia sp. 223 | Otu073 | 0.2734 | 0.1928 | -0.1598 | -0.2005 | -0.1630 | 0.1010 | 0.3085 | -0.1507 | -0.3338 | -0.2216 | -0.2341 |
| Leptotrichia unclassified | Otu005 | 0.3503 | 0.0997 | -0.1296 | -0.3446 | -0.3124 | 0.0321 | 0.2222 | -0.3287 | -0.4049 | -0.3225 | -0.3160 |
| Megasphaera micronuciformis 122 | Otu092 | 0.1212 | 0.1394 | -0.1334 | -0.1478 | -0.1520 | 0.0234 | 0.1249 | -0.1159 | -0.1723 | -0.1568 | -0.2563 |
| Moraxella catarrhalis 833 | Otu170 | 0.0378 | -0.0714 | 0.1980 | -0.0650 | -0.2163 | -0.1366 | 0.0902 | -0.0689 | -0.1532 | -0.1852 | -0.1523 |
| Neisseria oralis 014 | Otu074 | 0.0580 | -0.1013 | -0.0773 | -0.0633 | -0.1538 | -0.2563 | -0.1283 | -0.0035 | -0.1850 | -0.1692 | 0.1270 |
| Pasteurellaceae_unclassified | Otu019 | -0.0575 | -0.2647 | 0.1229 | 0.2069 | -0.1038 | -0.2806 | -0.1652 | 0.1479 | 0.2439 | 0.0769 | 0.3008 |
| Peptostreptococcaceae_[XI]_unclassified | Otu046 | 0.0273 | -0.2437 | 0.2467 | -0.1661 | -0.1361 | -0.1797 | -0.0295 | 0.0444 | 0.2063 | -0.1041 | 0.1820 |
| Peptostreptococcaceae_[XIII]_unclassified | Otu067 | -0.1163 | 0.0916 | 0.2540 | 0.2656 | 0.1392 | 0.0506 | -0.1462 | 0.1168 | 0.3339 | 0.0384 | 0.2515 |
| Porphyromonadaceae | Otu039 | -0.3096 | -0.1237 | 0.1268 | 0.4418 | 0.1565 | -0.1288 | -0.3310 | 0.3650 | 0.2391 | 0.3180 | 0.2260 |
| Porphyromonas endodontalis 273 | Otu033 | -0.2011 | -0.1923 | 0.1658 | 0.1397 | 0.0764 | -0.0795 | 0.0823 | 0.2004 | 0.1506 | 0.0022 | 0.3117 |
| Porphyromonas gingivalis 619 | Otu021 | -0.2219 | -0.0198 | 0.0676 | 0.2804 | 0.2283 | 0.0875 | -0.0649 | 0.3078 | 0.2006 | -0.0824 | 0.1516 |
| Porphyromonas sp. 279 | Otu022 | 0.0786 | 0.0421 | 0.1590 | 0.1663 | -0.0260 | -0.0485 | -0.0692 | 0.0580 | -0.2457 | -0.0496 | 0.0394 |
| Prevotella denticola 291 | Otu116 | 0.1424 | 0.0588 | -0.0949 | -0.1578 | -0.1976 | -0.0229 | 0.1469 | -0.1190 | -0.2015 | -0.1852 | -0.2362 |
| Prevotella enoeca 600 | Otu023 | -0.0594 | -0.2854 | 0.2521 | -0.0918 | -0.0597 | -0.1080 | 0.1982 | 0.1145 | -0.1236 | -0.2686 | -0.0519 |
| Prevotella fusca 782 | Otu082 | -0.0635 | 0.1828 | 0.0644 | -0.0398 | 0.2627 | 0.5040 | -0.3704 | 0.0752 | 0.3142 | 0.2299 | -0.0977 |
| Prevotella intermedia 643 | Otu062 | -0.3240 | -0.1923 | -0.1045 | 0.1077 | 0.0379 | -0.1516 | -0.1113 | 0.0545 | 0.2075 | 0.1940 | 0.1595 |
| Prevotella sp. 304 | Otu056 | -0.1916 | -0.0566 | 0.0102 | -0.0248 | 0.1682 | 0.3982 | -0.0263 | 0.2810 | 0.2295 | 0.1169 | 0.2155 |
| Prevotella sp. 311 | Otu040 | 0.0464 | -0.0148 | -0.2702 | -0.1745 | -0.2763 | -0.0751 | 0.2712 | -0.1624 | -0.2188 | -0.1524 | -0.2013 |
| Prevotella sp. 313 | Otu059 | -0.3700 | -0.0332 | -0.2907 | 0.0611 | 0.1438 | 0.0067 | -0.1860 | 0.0238 | 0.0948 | 0.2001 | -0.1010 |
| Prevotella sp. 317 | Otu053 | 0.2023 | 0.2220 | -0.1183 | -0.2489 | -0.2692 | 0.0358 | 0.2168 | -0.3026 | -0.3705 | -0.2903 | -0.3259 |
| Prevotella sp. 443 | Otu102 | -0.0025 | -0.0727 | -0.1352 | -0.1455 | 0.1437 | 0.1056 | -0.2313 | -0.0417 | 0.1598 | 0.1679 | 0.1960 |
| Prevotella sp. 526 | Otu013 | -0.1801 | -0.1220 | 0.0970 | -0.1371 | -0.0660 | 0.0497 | -0.0941 | 0.0031 | 0.1367 | -0.0070 | -0.0625 |
| Prevotella sp. 820 | Otu041 | -0.0250 | 0.0969 | -0.2312 | -0.0121 | -0.0339 | 0.2221 | 0.0480 | -0.0812 | 0.1031 | -0.0001 | -0.0476 |
| Prevotella unclassified | Otu008 | 0.1226 | 0.1896 | -0.0091 | -0.0599 | 0.1789 | 0.3037 | 0.2311 | 0.0520 | -0.0928 | 0.1943 | -0.0184 |
| Pyramidobacter piscolens 357 | Otu050 | 0.0587 | 0.2192 | 0.0396 | -0.0872 | 0.0538 | 0.3001 | -0.1582 | -0.1362 | 0.2225 | 0.0288 | -0.1102 |
| Selenomonas sputigena 151 | Otu011 | 0.3630 | 0.1061 | -0.2399 | -0.1891 | 0.0281 | 0.2897 | -0.0071 | -0.1142 | 0.0195 | 0.0232 | -0.0185 |
| Selenomonas unclassified | Otu004 | 0.2733 | -0.1719 | 0.0716 | -0.2530 | -0.1357 | 0.1040 | 0.1627 | -0.1177 | -0.1243 | -0.2317 | -0.0022 |
| SR1_[G-1] sp. 345 | Otu028 | -0.0771 | 0.0560 | 0.0490 | -0.0617 | 0.1843 | 0.3844 | -0.1080 | 0.0726 | 0.1779 | 0.1376 | 0.1676 |
| Streptococcus | Otu007 | 0.0046 | 0.2033 | -0.1042 | -0.0149 | 0.1659 | 0.0421 | 0.0252 | -0.0262 | 0.0361 | 0.2231 | -0.0271 |
| Streptococcus parasanguinis II 411 | Otu064 | -0.1062 | -0.0191 | -0.2192 | 0.0141 | -0.0765 | -0.2535 | -0.0190 | -0.0945 | -0.2357 | 0.0162 | -0.1891 |
| Streptococcus sp. 058 | Otu024 | 0.1116 | 0.0513 | -0.0147 | 0.1260 | 0.1411 | 0.0453 | 0.0294 | 0.0916 | -0.1962 | 0.1544 | 0.0762 |
| Treponema denticola 584 | Otu018 | -0.0142 | -0.0250 | -0.0333 | -0.1709 | -0.0282 | 0.0755 | 0.1409 | -0.0461 | -0.1110 | -0.1458 | -0.0344 |
| Treponema maltophilum 664 | Otu026 | -0.0063 | -0.3028 | 0.2082 | 0.1183 | -0.0208 | -0.1907 | -0.0902 | 0.1685 | 0.2105 | 0.0878 | 0.2222 |
| Treponema socranskii 769 | Otu060 | 0.1772 | -0.2770 | -0.0810 | -0.2975 | -0.2807 | -0.1638 | 0.2288 | -0.2143 | -0.1607 | -0.1649 | 0.0592 |
| Treponema sp. 246 | Otu104 | -0.0831 | 0.0478 | 0.0216 | 0.0964 | -0.0641 | -0.0563 | -0.1581 | 0.0567 | -0.0309 | -0.1329 | -0.0922 |
| Treponema unclassified | Otu002 | 0.1020 | 0.0031 | 0.0076 | 0.0599 | 0.0795 | 0.2161 | -0.1175 | 0.0921 | 0.0623 | 0.0041 | 0.2607 |
| Veillonella dispar 160 | Otu058 | -0.4301 | -0.1859 | -0.2731 | 0.0594 | 0.0472 | -0.2958 | -0.2294 | -0.1372 | 0.1422 | 0.2726 | -0.1703 |
| Veillonella parvula 161 | Otu010 | -0.1631 | 0.2581 | -0.3035 | 0.0097 | 0.1758 | -0.1593 | 0.1078 | -0.1867 | 0.1419 | 0.1756 | -0.1104 |
| Veillonella unclassified | Otu006 | 0.0533 | 0.1440 | -0.1468 | 0.0878 | 0.2758 | 0.2237 | 0.1680 | 0.0961 | -0.0360 | 0.2935 | 0.0345 |
| Veillonellaceae_[G-1] | Otu025 | -0.1638 | -0.2098 | 0.4364 | 0.3672 | 0.1996 | -0.0015 | -0.3392 | 0.2723 | 0.2395 | 0.3187 | 0.1795 |
| Veillonellaceae_[G-1] sp. 155 | Otu048 | 0.1825 | 0.2980 | -0.2563 | -0.2609 | -0.3783 | -0.0006 | 0.2408 | -0.2664 | -0.3963 | -0.3557 | -0.3731 |
|  |  | **RB1** | **ATG16L2** | **ATG4C** | **ATG4D** | **CALCOCO2** | **CXCR4** | **EIF2AK3** | **GCN2** | **GABARAPL2** | **PLIN2** | **ATG3** |
| A. actinomycetemcomitans 531 | Otu017 | 0.0670 | -0.1707 | -0.0479 | 0.1413 | 0.0519 | -0.0049 | -0.0456 | 0.1519 | 0.0520 | -0.0704 | 0.0578 |
| Aggregatibacter unclassified | Otu069 | -0.1645 | 0.0932 | 0.0183 | -0.2457 | 0.1600 | 0.1282 | 0.0562 | 0.0419 | -0.1663 | 0.1130 | -0.1945 |
| Bacteria_unclassified | Otu003 | 0.3481 | 0.1219 | 0.1711 | 0.1155 | 0.1030 | -0.0759 | 0.0324 | 0.0566 | -0.2568 | 0.0541 | -0.0981 |
| Bacteroidetes_unclassified | Otu020 | -0.0504 | 0.1284 | -0.0879 | 0.1724 | -0.1990 | -0.0276 | -0.0651 | -0.0128 | -0.3047 | -0.0357 | -0.3310 |
| Capnocytophaga unclassified | Otu015 | -0.0857 | 0.0413 | -0.0978 | 0.2580 | -0.1593 | -0.3617 | -0.3240 | -0.0081 | 0.3679 | -0.1259 | 0.1967 |
| Catonella morbi 165 | Otu014 | -0.3437 | -0.0946 | -0.1086 | -0.0108 | 0.0627 | -0.0273 | -0.0128 | 0.1128 | 0.0456 | 0.0158 | -0.2040 |
| Chloroflexi_[G-1] sp. 439 | Otu030 | 0.0489 | 0.1437 | -0.0473 | 0.0499 | 0.1309 | 0.1099 | 0.0497 | 0.0790 | -0.3063 | 0.1358 | -0.0841 |
| Desulfobulbus sp. 041 | Otu084 | 0.0155 | -0.0531 | 0.0322 | 0.2188 | 0.0710 | -0.2681 | -0.3461 | 0.1559 | 0.0378 | -0.2363 | -0.2228 |
| Eubacterium infirmum 105 | Otu037 | -0.1699 | -0.0651 | -0.0209 | -0.1864 | 0.0081 | 0.1302 | 0.0115 | -0.1459 | 0.1210 | -0.0421 | -0.0847 |
| Filifactor alocis 539 | Otu063 | 0.1975 | 0.1887 | 0.2039 | -0.2041 | 0.1999 | 0.1962 | 0.2103 | 0.0939 | -0.4820 | 0.3343 | -0.0169 |
| Fretibacterium fastidiosum 363 | Otu027 | 0.0136 | 0.0935 | 0.0419 | -0.0267 | 0.0969 | -0.0562 | -0.1562 | 0.3594 | -0.3032 | -0.0105 | -0.1631 |
| Fretibacterium sp. 361 | Otu009 | -0.0874 | 0.2235 | 0.0374 | -0.0809 | 0.0700 | 0.0924 | -0.1388 | 0.2655 | -0.4613 | 0.1135 | -0.2507 |
| Fretibacterium unclassified | Otu012 | 0.0915 | -0.2101 | 0.0207 | 0.2318 | -0.0191 | -0.2499 | -0.1477 | -0.1494 | 0.5499 | -0.1990 | 0.2312 |
| Fusobacterium sp. 203 | Otu032 | -0.0265 | 0.0993 | 0.1857 | 0.2161 | 0.2106 | -0.2462 | -0.1074 | 0.2032 | 0.2643 | -0.2046 | -0.1280 |
| Fusobacterium unclassified | Otu001 | -0.0928 | -0.0233 | -0.0895 | -0.1584 | -0.1192 | 0.0172 | -0.0645 | -0.0979 | -0.0106 | 0.2153 | 0.1379 |
| Gemella morbillorum 046 | Otu045 | -0.0409 | -0.0823 | -0.1636 | -0.0693 | -0.2499 | -0.0476 | -0.0606 | -0.1795 | 0.0329 | 0.1649 | 0.0896 |
| Haemophilus sp. 035 | Otu093 | -0.0489 | -0.2031 | -0.0999 | 0.0109 | -0.1788 | -0.0836 | -0.1369 | -0.1859 | 0.0485 | -0.0229 | -0.1030 |
| Leptotrichia sp. 223 | Otu073 | 0.1192 | -0.0847 | 0.1335 | 0.2807 | 0.1414 | -0.3230 | -0.1359 | -0.1719 | 0.5747 | -0.2424 | 0.2111 |
| Leptotrichia unclassified | Otu005 | -0.1698 | -0.1670 | -0.1039 | 0.4069 | -0.0891 | -0.3322 | -0.2731 | 0.1222 | 0.2927 | -0.3105 | -0.1233 |
| Megasphaera micronuciformis 122 | Otu092 | 0.1865 | -0.0246 | 0.0275 | 0.0598 | 0.0483 | -0.1236 | -0.1045 | -0.0582 | 0.1404 | -0.0603 | -0.0489 |
| Moraxella catarrhalis 833 | Otu170 | -0.1208 | -0.0840 | -0.1049 | -0.0268 | -0.1759 | -0.1366 | -0.2120 | -0.1259 | 0.2613 | -0.1314 | -0.0467 |
| Neisseria oralis 014 | Otu074 | -0.1229 | 0.0284 | -0.2044 | 0.0947 | 0.0492 | -0.1384 | -0.1555 | 0.2563 | -0.0174 | -0.1013 | -0.1290 |
| Pasteurellaceae_unclassified | Otu019 | -0.1387 | 0.1447 | -0.1032 | -0.1756 | -0.1829 | 0.0604 | 0.0202 | -0.0415 | -0.3258 | 0.1710 | -0.0525 |
| Peptostreptococcaceae_[XI]_unclassified | Otu046 | -0.1666 | 0.1673 | -0.0396 | 0.1917 | -0.1425 | -0.0594 | -0.1888 | 0.0043 | -0.1818 | -0.0877 | -0.3154 |
| Peptostreptococcaceae_[XIII]_unclassified | Otu067 | 0.1938 | -0.0063 | 0.1490 | -0.1119 | 0.1686 | 0.0343 | 0.0227 | 0.2705 | -0.4736 | 0.1766 | -0.1696 |
| Porphyromonadaceae | Otu039 | 0.0524 | 0.3066 | -0.0351 | -0.4453 | -0.0040 | 0.2054 | 0.2804 | 0.0321 | -0.2433 | 0.5551 | 0.3403 |
| Porphyromonas endodontalis 273 | Otu033 | -0.1662 | 0.1001 | 0.0013 | -0.1675 | 0.2253 | 0.1418 | 0.0870 | 0.1292 | -0.0900 | 0.1817 | 0.0291 |
| Porphyromonas gingivalis 619 | Otu021 | 0.1140 | 0.1395 | 0.2718 | -0.2275 | 0.1703 | -0.0130 | 0.0048 | 0.1939 | -0.2265 | 0.2535 | 0.1814 |
| Porphyromonas sp. 279 | Otu022 | -0.0799 | -0.1377 | -0.1415 | 0.0319 | -0.1710 | -0.0931 | -0.1380 | -0.1265 | 0.0496 | 0.0197 | -0.0808 |
| Prevotella denticola 291 | Otu116 | 0.1165 | -0.0183 | -0.0230 | 0.0795 | 0.0381 | -0.1458 | -0.1543 | -0.0092 | 0.1303 | -0.0781 | -0.0917 |
| Prevotella enoeca 600 | Otu023 | -0.1969 | 0.1594 | 0.2000 | -0.0224 | 0.2186 | -0.0728 | -0.1078 | 0.1440 | 0.2576 | -0.0695 | -0.0948 |
| Prevotella fusca 782 | Otu082 | 0.2106 | 0.0559 | 0.1716 | -0.1413 | 0.0918 | 0.1411 | 0.1630 | -0.2915 | -0.0032 | 0.1217 | -0.0484 |
| Prevotella intermedia 643 | Otu062 | 0.0109 | 0.0221 | 0.0520 | -0.2359 | -0.0166 | 0.2014 | 0.3002 | -0.1201 | -0.1782 | 0.0594 | 0.1653 |
| Prevotella sp. 304 | Otu056 | -0.2739 | 0.0030 | 0.0397 | -0.0007 | 0.0239 | 0.2068 | 0.1148 | -0.1268 | -0.0195 | 0.0651 | 0.0403 |
| Prevotella sp. 311 | Otu040 | 0.0751 | -0.0752 | -0.0511 | 0.0394 | 0.0556 | -0.0903 | -0.0613 | -0.1281 | 0.1685 | -0.1780 | 0.0525 |
| Prevotella sp. 313 | Otu059 | -0.1376 | -0.1428 | 0.0497 | -0.1557 | -0.1233 | 0.1133 | 0.1645 | -0.3545 | -0.0177 | 0.0797 | 0.1129 |
| Prevotella sp. 317 | Otu053 | -0.0155 | -0.1175 | 0.0269 | 0.3269 | -0.0392 | -0.3792 | -0.2389 | 0.1183 | 0.3359 | -0.2646 | -0.0151 |
| Prevotella sp. 443 | Otu102 | -0.0273 | -0.0386 | -0.2475 | -0.0296 | -0.1569 | 0.1109 | 0.1364 | -0.1654 | -0.1285 | 0.0102 | -0.1207 |
| Prevotella sp. 526 | Otu013 | -0.0358 | -0.0463 | -0.1604 | -0.2602 | -0.1760 | 0.0058 | -0.0520 | -0.0998 | -0.0917 | -0.0557 | -0.1397 |
| Prevotella sp. 820 | Otu041 | 0.1318 | -0.1757 | 0.1339 | -0.0853 | 0.0387 | -0.0625 | 0.1156 | -0.2794 | 0.0447 | -0.0602 | 0.1122 |
| Prevotella unclassified | Otu008 | 0.0035 | -0.0115 | 0.1699 | 0.0481 | 0.3301 | 0.2884 | 0.2773 | 0.1230 | 0.2655 | -0.0313 | 0.1076 |
| Pyramidobacter piscolens 357 | Otu050 | 0.3030 | 0.0710 | 0.1148 | 0.0859 | 0.0207 | -0.0936 | -0.0286 | 0.0263 | -0.1631 | -0.0079 | -0.1014 |
| Selenomonas sputigena 151 | Otu011 | 0.1410 | -0.1178 | 0.0223 | 0.1173 | -0.0753 | -0.1757 | 0.0476 | 0.0361 | 0.0512 | -0.0757 | -0.0194 |
| Selenomonas unclassified | Otu004 | -0.2153 | 0.0636 | 0.0343 | 0.1631 | 0.1796 | -0.1836 | -0.1332 | 0.2313 | 0.1191 | -0.1912 | -0.2808 |
| SR1_[G-1] sp. 345 | Otu028 | 0.1606 | 0.0332 | 0.0434 | -0.1010 | 0.0616 | 0.1205 | 0.1210 | 0.0521 | -0.1643 | -0.0165 | 0.0671 |
| Streptococcus | Otu007 | -0.1154 | -0.0791 | -0.0482 | 0.0036 | 0.1300 | 0.3259 | 0.1952 | -0.1231 | 0.1467 | -0.0466 | -0.0073 |
| Streptococcus parasanguinis II 411 | Otu064 | -0.2793 | -0.1888 | -0.1667 | -0.0022 | -0.1516 | 0.0049 | -0.0770 | -0.2416 | 0.2173 | -0.0272 | 0.0541 |
| Streptococcus sp. 058 | Otu024 | -0.0838 | -0.1959 | -0.0861 | 0.0346 | 0.0208 | 0.0579 | 0.0518 | -0.0217 | 0.0225 | 0.0200 | -0.0264 |
| Treponema denticola 584 | Otu018 | -0.0600 | -0.1414 | -0.1301 | 0.2665 | -0.0323 | -0.0540 | -0.1067 | 0.0372 | 0.1215 | -0.1657 | 0.0429 |
| Treponema maltophilum 664 | Otu026 | -0.1329 | 0.2518 | -0.0598 | -0.0556 | -0.1936 | 0.0435 | -0.0283 | 0.0886 | -0.2222 | 0.1215 | 0.0279 |
| Treponema socranskii 769 | Otu060 | -0.2985 | -0.1272 | -0.3108 | 0.2047 | -0.0477 | -0.2159 | -0.1627 | -0.1575 | 0.2407 | -0.3052 | -0.1518 |
| Treponema sp. 246 | Otu104 | 0.1148 | -0.1030 | 0.0880 | 0.1232 | 0.0088 | 0.0131 | -0.1211 | 0.0942 | -0.0975 | 0.0231 | 0.1534 |
| Treponema unclassified | Otu002 | 0.1856 | -0.1067 | 0.0540 | 0.1874 | -0.1102 | -0.0066 | 0.0066 | 0.1005 | -0.1418 | -0.0965 | 0.1635 |
| Veillonella dispar 160 | Otu058 | -0.2558 | -0.1101 | -0.0940 | -0.1972 | -0.2356 | 0.2107 | 0.1309 | -0.3395 | -0.0961 | -0.0007 | -0.0009 |
| Veillonella parvula 161 | Otu010 | 0.0652 | 0.0737 | 0.0841 | -0.1302 | 0.1447 | 0.3341 | 0.2569 | -0.0713 | 0.0195 | -0.0645 | 0.0502 |
| Veillonella unclassified | Otu006 | 0.1322 | -0.0081 | 0.2069 | -0.0357 | 0.2064 | 0.1852 | 0.3270 | 0.2073 | 0.0468 | 0.0881 | 0.1794 |
| Veillonellaceae_[G-1] | Otu025 | -0.0165 | 0.4113 | 0.0944 | -0.3979 | 0.0156 | 0.2005 | 0.1747 | 0.1299 | -0.4133 | 0.3592 | -0.0222 |
| Veillonellaceae_[G-1] sp. 155 | Otu048 | 0.0685 | -0.1742 | -0.0717 | 0.3472 | -0.2561 | -0.3905 | -0.3421 | -0.0158 | 0.3006 | -0.2728 | 0.1742 |
|  |  | **ATG5** | **ATG7** | **BAK1** | **CTSL2** | **EPAS1** | **FAS** | **LAMP2** | **PRKCQ** | **VAMP8** | **BAD** | **CASP8** |
| A. actinomycetemcomitans 531 | Otu017 | 0.0543 | 0.0033 | 0.0364 | 0.0327 | -0.0346 | 0.1367 | 0.0041 | -0.1161 | -0.1161 | 0.0959 | -0.0933 |
| Aggregatibacter unclassified | Otu069 | 0.0815 | 0.1310 | -0.1352 | -0.1525 | 0.2105 | -0.1028 | -0.1716 | 0.0958 | 0.0958 | -0.0419 | 0.0051 |
| Bacteria_unclassified | Otu003 | 0.1406 | 0.1664 | -0.0233 | -0.0616 | 0.0675 | 0.0496 | 0.0277 | 0.1594 | 0.1594 | -0.1180 | -0.1187 |
| Bacteroidetes_unclassified | Otu020 | -0.0727 | 0.1449 | -0.0964 | -0.0069 | 0.1127 | -0.0259 | -0.1185 | 0.0486 | 0.0486 | 0.0791 | -0.1321 |
| Capnocytophaga unclassified | Otu015 | -0.1956 | 0.0998 | -0.1847 | 0.2032 | -0.0742 | 0.2743 | 0.3879 | -0.0995 | -0.0995 | -0.1545 | -0.1209 |
| Catonella morbi 165 | Otu014 | 0.0795 | 0.1156 | 0.0992 | -0.0611 | 0.0980 | -0.1020 | -0.1293 | -0.0454 | -0.0454 | -0.0866 | 0.0192 |
| Chloroflexi_[G-1] sp. 439 | Otu030 | 0.0183 | 0.1234 | -0.0162 | -0.1269 | 0.1632 | -0.0652 | -0.1150 | 0.1637 | 0.1637 | 0.0092 | -0.0187 |
| Desulfobulbus sp. 041 | Otu084 | -0.2450 | 0.0620 | -0.3776 | 0.2151 | 0.0896 | 0.1710 | 0.1553 | -0.1825 | -0.1825 | -0.4616 | -0.2704 |
| Eubacterium infirmum 105 | Otu037 | 0.0155 | -0.0730 | -0.0131 | -0.0690 | -0.1782 | -0.0399 | -0.0109 | -0.0311 | -0.0311 | -0.0356 | -0.1488 |
| Filifactor alocis 539 | Otu063 | 0.2196 | 0.0837 | 0.2194 | -0.2935 | 0.3264 | -0.1134 | -0.2631 | -0.0655 | -0.0655 | 0.0356 | 0.0852 |
| Fretibacterium fastidiosum 363 | Otu027 | -0.1028 | -0.1575 | 0.0266 | -0.1980 | 0.2640 | 0.1931 | -0.1694 | 0.0119 | 0.0119 | -0.1961 | -0.0252 |
| Fretibacterium sp. 361 | Otu009 | -0.1266 | -0.1835 | 0.0221 | -0.3216 | 0.4265 | 0.1424 | -0.2959 | 0.1114 | 0.1114 | -0.2649 | -0.0439 |
| Fretibacterium unclassified | Otu012 | -0.0326 | 0.2289 | -0.2235 | 0.5315 | -0.2848 | 0.0466 | 0.3858 | -0.1813 | -0.1813 | -0.0424 | -0.0505 |
| Fusobacterium sp. 203 | Otu032 | -0.2172 | 0.0518 | -0.2367 | 0.1542 | 0.0497 | 0.1497 | 0.2588 | -0.1137 | -0.1137 | -0.2944 | -0.1044 |
| Fusobacterium unclassified | Otu001 | 0.0487 | -0.0933 | 0.2789 | -0.0927 | 0.1166 | -0.0217 | -0.1725 | -0.1677 | -0.1677 | 0.1730 | 0.1760 |
| Gemella morbillorum 046 | Otu045 | 0.0603 | -0.0019 | 0.2625 | -0.0461 | -0.0322 | 0.0714 | -0.1148 | -0.0277 | -0.0277 | 0.1263 | 0.1619 |
| Haemophilus sp. 035 | Otu093 | -0.0027 | -0.0386 | 0.0768 | 0.0160 | -0.0210 | -0.0008 | -0.1667 | -0.1414 | -0.1414 | 0.1526 | 0.0030 |
| Leptotrichia sp. 223 | Otu073 | -0.1318 | 0.2400 | -0.2340 | 0.3950 | -0.1379 | 0.2092 | 0.5632 | -0.1125 | -0.1125 | -0.0557 | -0.1733 |
| Leptotrichia unclassified | Otu005 | -0.1359 | 0.2634 | -0.2934 | 0.2347 | -0.1136 | 0.1936 | 0.3869 | -0.0728 | -0.0728 | -0.2232 | -0.2002 |
| Megasphaera micronuciformis 122 | Otu092 | -0.1489 | -0.0373 | -0.1717 | 0.1693 | -0.1382 | 0.0618 | 0.2127 | -0.0999 | -0.0999 | 0.1625 | -0.1927 |
| Moraxella catarrhalis 833 | Otu170 | -0.2146 | -0.1493 | -0.1368 | -0.0182 | -0.1136 | 0.0910 | 0.1069 | -0.0872 | -0.0872 | -0.2018 | -0.0908 |
| Neisseria oralis 014 | Otu074 | 0.0849 | -0.0593 | -0.0299 | 0.0488 | 0.1086 | -0.1357 | -0.0859 | -0.1388 | -0.1388 | -0.0508 | -0.0554 |
| Pasteurellaceae_unclassified | Otu019 | -0.0461 | -0.1511 | 0.0211 | -0.2958 | 0.1569 | -0.1619 | -0.2502 | 0.0957 | 0.0957 | 0.0363 | 0.0711 |
| Peptostreptococcaceae_[XI]_unclassified | Otu046 | -0.1691 | 0.0221 | -0.2247 | 0.0208 | 0.1943 | -0.0100 | -0.0568 | 0.0066 | 0.0066 | -0.0265 | -0.1699 |
| Peptostreptococcaceae_[XIII]_unclassified | Otu067 | 0.1950 | 0.0473 | 0.0995 | -0.2684 | 0.3588 | 0.1607 | -0.1611 | 0.0012 | 0.0012 | -0.0842 | -0.0226 |
| Porphyromonadaceae | Otu039 | 0.2702 | -0.1371 | 0.2908 | -0.2793 | 0.0922 | -0.2120 | -0.1971 | 0.2389 | 0.2389 | 0.0933 | 0.3143 |
| Porphyromonas endodontalis 273 | Otu033 | -0.0351 | -0.0929 | 0.0537 | -0.1838 | 0.2828 | -0.1311 | -0.0686 | -0.1723 | -0.1723 | 0.0089 | -0.0034 |
| Porphyromonas gingivalis 619 | Otu021 | 0.0167 | -0.0671 | 0.1806 | -0.2286 | 0.3544 | 0.0588 | -0.1100 | -0.1865 | -0.1865 | -0.0439 | 0.0842 |
| Porphyromonas sp. 279 | Otu022 | -0.0180 | -0.0024 | 0.1275 | 0.0396 | 0.0206 | 0.0602 | -0.1213 | -0.0639 | -0.0639 | 0.1016 | 0.0761 |
| Prevotella denticola 291 | Otu116 | -0.2025 | -0.0433 | -0.1830 | 0.1641 | -0.1023 | 0.0613 | 0.2000 | -0.1386 | -0.1386 | 0.1511 | -0.2111 |
| Prevotella enoeca 600 | Otu023 | -0.2468 | -0.0237 | -0.1963 | 0.2372 | 0.1751 | -0.0442 | 0.1291 | -0.2043 | -0.2043 | -0.2139 | -0.0738 |
| Prevotella fusca 782 | Otu082 | 0.3003 | 0.2368 | 0.2502 | 0.0919 | -0.1397 | -0.1051 | -0.0210 | 0.0982 | 0.0982 | 0.0681 | 0.0304 |
| Prevotella intermedia 643 | Otu062 | 0.1904 | 0.0717 | 0.0099 | -0.1371 | -0.1438 | -0.2627 | -0.2227 | 0.1055 | 0.1055 | 0.1017 | 0.2503 |
| Prevotella sp. 304 | Otu056 | -0.0347 | 0.1259 | 0.2769 | -0.1385 | 0.1732 | -0.1033 | -0.1093 | -0.1168 | -0.1168 | 0.0452 | -0.0242 |
| Prevotella sp. 311 | Otu040 | -0.2462 | 0.0010 | -0.2098 | 0.1830 | -0.1666 | -0.0588 | 0.1771 | -0.1993 | -0.1993 | 0.1745 | -0.1676 |
| Prevotella sp. 313 | Otu059 | 0.2414 | -0.1354 | 0.1137 | -0.1734 | -0.2682 | -0.2676 | -0.2493 | -0.0131 | -0.0131 | 0.2655 | -0.1099 |
| Prevotella sp. 317 | Otu053 | -0.0961 | 0.1174 | -0.2105 | 0.2122 | -0.1183 | 0.2301 | 0.4758 | -0.2263 | -0.2263 | -0.2275 | -0.1674 |
| Prevotella sp. 443 | Otu102 | 0.2107 | 0.1150 | 0.1034 | -0.0163 | -0.1233 | -0.0702 | -0.1655 | 0.2754 | 0.2754 | 0.2161 | 0.0850 |
| Prevotella sp. 526 | Otu013 | -0.0020 | -0.1014 | -0.0667 | -0.0838 | -0.1355 | -0.0144 | 0.0415 | 0.0963 | 0.0963 | 0.0373 | -0.0864 |
| Prevotella sp. 820 | Otu041 | 0.1062 | 0.2222 | 0.0271 | -0.0046 | -0.1218 | -0.0249 | 0.0496 | 0.0116 | 0.0116 | 0.0096 | -0.0342 |
| Prevotella unclassified | Otu008 | -0.0219 | 0.1441 | 0.0473 | 0.0965 | -0.0448 | 0.1094 | 0.1862 | 0.1254 | 0.1254 | -0.2449 | 0.2038 |
| Pyramidobacter piscolens 357 | Otu050 | 0.1618 | 0.2150 | -0.0282 | 0.0434 | -0.0613 | -0.0489 | 0.0517 | -0.0499 | -0.0499 | -0.0752 | -0.2186 |
| Selenomonas sputigena 151 | Otu011 | 0.1129 | -0.0648 | 0.0703 | 0.0596 | -0.1967 | 0.0010 | 0.1721 | 0.0837 | 0.0837 | 0.0419 | 0.0289 |
| Selenomonas unclassified | Otu004 | -0.1824 | -0.0823 | -0.1828 | 0.2044 | 0.0972 | -0.0220 | 0.1364 | -0.0424 | -0.0424 | -0.2091 | -0.1135 |
| SR1_[G-1] sp. 345 | Otu028 | 0.0540 | 0.0202 | 0.1155 | -0.1513 | 0.0239 | 0.1492 | 0.0966 | 0.0420 | 0.0420 | 0.0458 | -0.0012 |
| Streptococcus | Otu007 | 0.1100 | 0.2508 | 0.0460 | 0.0571 | -0.0451 | -0.1191 | -0.1368 | 0.2412 | 0.2412 | -0.0222 | 0.1479 |
| Streptococcus parasanguinis II 411 | Otu064 | 0.1138 | 0.0806 | 0.0229 | 0.0154 | -0.2093 | -0.1571 | -0.1302 | -0.0835 | -0.0835 | 0.1488 | -0.0764 |
| Streptococcus sp. 058 | Otu024 | 0.0853 | 0.1712 | 0.1860 | -0.0887 | 0.0682 | 0.0318 | -0.1733 | -0.0892 | -0.0892 | 0.0911 | 0.1346 |
| Treponema denticola 584 | Otu018 | -0.1012 | 0.1164 | 0.0498 | 0.1222 | 0.0285 | 0.1802 | 0.0839 | -0.0694 | -0.0694 | 0.0834 | -0.0537 |
| Treponema maltophilum 664 | Otu026 | -0.1304 | -0.2022 | 0.1076 | -0.2239 | 0.1779 | 0.1076 | -0.0835 | 0.1775 | 0.1775 | -0.1577 | 0.2451 |
| Treponema socranskii 769 | Otu060 | -0.2107 | -0.1494 | -0.2130 | 0.2890 | -0.0874 | -0.1411 | 0.2508 | -0.1291 | -0.1291 | -0.0625 | -0.1218 |
| Treponema sp. 246 | Otu104 | -0.0131 | 0.0340 | 0.0907 | -0.0569 | 0.0265 | 0.2762 | -0.0557 | 0.0240 | 0.0240 | 0.1267 | -0.0261 |
| Treponema unclassified | Otu002 | 0.0095 | 0.2103 | 0.1799 | -0.1245 | 0.0981 | 0.2710 | -0.0339 | -0.0538 | -0.0538 | 0.0002 | 0.0105 |
| Veillonella dispar 160 | Otu058 | 0.2041 | -0.2620 | 0.0382 | -0.1553 | -0.3432 | -0.3808 | -0.3522 | 0.0130 | 0.0130 | 0.2649 | -0.1046 |
| Veillonella parvula 161 | Otu010 | 0.0474 | -0.1622 | -0.1784 | 0.0551 | -0.1038 | -0.2043 | -0.1070 | 0.2648 | 0.2648 | -0.0384 | 0.0999 |
| Veillonella unclassified | Otu006 | 0.1641 | 0.2304 | 0.0881 | -0.0966 | -0.0081 | 0.0109 | -0.0161 | 0.1298 | 0.1298 | -0.1773 | 0.2863 |
| Veillonellaceae_[G-1] | Otu025 | 0.1136 | -0.1032 | 0.2739 | -0.2200 | 0.2982 | -0.0638 | -0.3132 | 0.1528 | 0.1528 | -0.1632 | 0.2753 |
| Veillonellaceae_[G-1] sp. 155 | Otu048 | -0.2291 | 0.0195 | -0.1214 | 0.0558 | -0.1922 | 0.3926 | 0.5183 | -0.2846 | -0.2846 | -0.0952 | -0.2189 |
